# Rapid start-up of carbon-free H_2_ production by ammonia oxidative decomposition over Co/Ce_0.5_Zr_0.5_O_2_ with microwave irradiation

**DOI:** 10.1016/j.isci.2024.110452

**Published:** 2024-07-03

**Authors:** Takahiro Matsunaga, Sachika Hayashi, Hiroshi Yamada, Katsutoshi Sato, Katsutoshi Nagaoka

**Affiliations:** 1 Department of Chemical Systems Engineering, Graduate School of Engineering, Nagoya University, Furo-cho, Chikusa-ku, Nagoya 464-8603, Japan; 2 Institute for Advanced Research Nagoya University, Furo-cho, Chikusa-ku, Nagoya 464-8603, Japan

**Keywords:** Chemistry, Chemical engineering, Materials science, Materials chemistry

## Abstract

Hydrogen is a promising combustion improver for use with ammonia fuels, but a cost-effective method for easily producing hydrogen from ammonia at a high rate has yet to be developed. Here, we show that microwave irradiation instantly triggers oxidative decomposition of ammonia over a Co/Ce_0.5_Zr_0.5_O_2_ catalyst to produce hydrogen at a high rate. The microwave irradiation rapidly heats the inside of the catalyst from room temperature to the catalytic auto-ignition temperature of ammonia, thus initiating exothermic oxidative decomposition of ammonia to produce hydrogen. This method provides a highly efficient means of producing hydrogen for potential use in a carbon-free, ammonia-fueled power generation process.

## Highlights

• Microwave irradiation rapidly triggers NH_3_ oxidative decomposition
• Microwave irradiation rapidly heats Co/Ce_0.5_Zr_0.5_O_2_ catalyst
• Co/Ce_0.5_Zr_0.5_O_2_ catalyst boosts H_2_ production from NH_3_ and O_2_
• NH_3_ oxidative decomposition can be triggered repeatedly over Co/Ce_0.5_Zr_0.5_O_2_

## Graphical abstract

**Figure:**
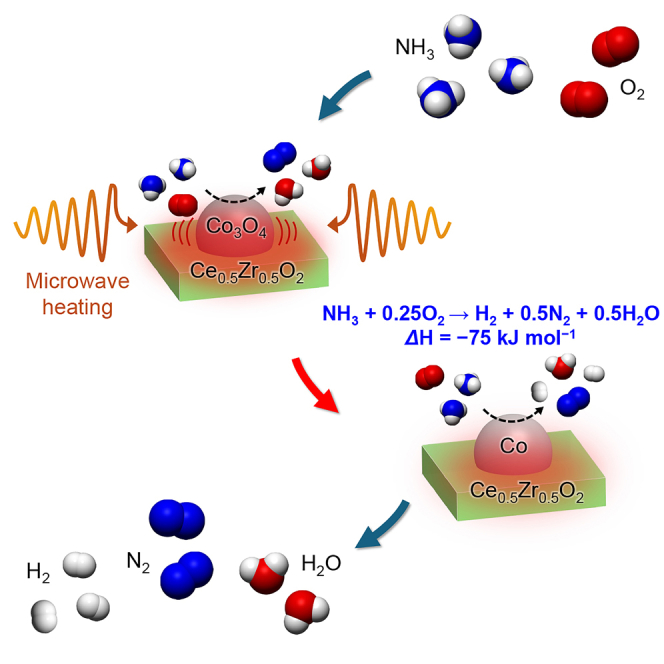

## Introduction

Recently, ammonia-fueled power generation processes have gained attention as potentially important means of realizing a carbon-neutral society.^1^^,^^2^^,^^3^^,^^4^ In such processes, ammonia is synthesized via the Haber-Bosch process from nitrogen from air and hydrogen produced by water electrolysis and photocatalytic water splitting.^5^^,^^6^^,^^7^^,^^8^ After liquefaction, the ammonia is transported in large quantities by ship to storage facilities at power plants, factories, or other locations equipped to use ammonia as a fuel. When used as a fuel, ammonia is decomposed to nitrogen and hydrogen, and the hydrogen is used for the generation of electricity. Importantly for mitigating global warming, this process does not emit CO_2_ because the ammonia molecule contains no carbon atoms. One of the challenges that remain for using ammonia for power generation is that although ammonia can be combusted in power plants, it has low flammability and is difficult to ignite.^9^^,^^10^ It has been reported that mixing ammonia with hydrogen can improve its combustion properties^11^^,^^12^; therefore, we hypothesized that the hydrogen produced during the catalytic decomposition of ammonia could be harnessed and used as a combustion improver. Thus, an efficient process to produce hydrogen easily, quickly, and at a high rate at any time without complicated procedures is required.

Conventional direct decomposition of ammonia (Equation 1) is an endothermic reaction.^13^^,^^14^ In 2023, Zheng et al.^15^ and Su et al.^16^ reviewed the state of the art of catalysts for this reaction. Among the different kinds of catalysts available, which include Ru, Fe, Co, or Ni-based, metal nitride, metal carbide, and amide-imide catalysts, supported Ru catalysts doped with strong alkaline substrates and with a high electrical conductivity and high surface area have shown excellent performance for ammonia decomposition. However, the fundamental issue remains that ammonia decomposition is endothermic, meaning that the catalyst must continue to be heated by an external heat source throughout the reaction, requiring a temperature of 400°C (99% equilibrium conversion of ammonia at 0.1 MPa) for ammonia consumption, as well as diffusion of heat throughout the catalyst particles. Also, it takes a long time to heat the reactor from room temperature to the temperature (≥400°C) at which sufficient equilibrium conversion is obtained, and large amounts of energy are consumed to heat the reactor.

Oxidative decomposition is a potential alternative to the conventional direct decomposition approach.^17^ Unlike direct decomposition, oxidative decomposition of ammonia (Equation 2) is an exothermic reaction. In this reaction, a low concentration of O_2_ is added to the ammonia as a reactant, exothermic ammonia combustion (Equation 3) occurs at the reactor inlet, and endothermic ammonia decomposition (Equation 1) occurs using the heat generated. Thus, after the oxidative decomposition of ammonia is started, the reaction proceeds without any further external heat input.

(Equation 1) $$\begin{array}{cc} {\text{NH}}_{3}\left(g\right)\to 1.5H_{2}\left(g\right)+0.5N_{2}\left(g\right) & \Delta H=+46\;\text{kJ}\;{\text{mol}}^{-1} \end{array}$$

(Equation 2) $$\begin{array}{cc} {\text{NH}}_{3}\left(g\right)+0.25O_{2}\left(g\right)\to H_{2}\left(g\right)+0.5\;N_{2}\left(g\right)+0.5H_{2}O\left(g\right) & \Delta H=-75\;\text{kJ}\;{\text{mol}}^{-1} \end{array}$$

(Equation 3) $$\begin{array}{cc} {\text{NH}}_{3}\left(g\right)+0.75O_{2}\left(g\right)\to 0.5N_{2}\left(g\right)+1.5H_{2}O\left(g\right) & \Delta H=-317\;\text{kJ}\;{\text{mol}}^{-1} \end{array}$$

The key to rapid initiation of oxidative decomposition of ammonia at room temperature is the ability to instantaneously heat the catalyst from room temperature to the catalytic auto-ignition temperature of ammonia. For this purpose, we have previously used a combination of three heat sources: heat generated by catalyst self-heating, heat generated by ammonia adsorption on the acid sites of pre-treated RuO_2_/γ-Al_2_O_3_ catalyst,^18^ and heat generated by oxidation of reduced Ru/Ce_0.5_Zr_0.5_O_2_ catalyst.^19^ With this approach, oxidative decomposition of ammonia is triggered at room temperature by supplying NH_3_ and O_2_ to these catalysts. However, despite the efficacy of the catalysts, sublimable RuO_4_ can only be produced using large-scale processes, and Ru is a rare precious metal; therefore, catalysts that do not require a sublimable compound or a precious metal are needed. However, a major issue with non-precious metal catalysts is their lower reducibility and higher auto-ignition temperature compared to precious metal catalysts. For example, Li et al. have reported that Co-Ni/Ce_0.5_Zr_0.5_O_2_ pre-treated in N_2_ at 300°C and left in an N_2_ atmosphere is able to initiate oxidative decomposition of ammonia at 100°C^20^; in their method, they used an electric furnace to maintain the temperature of the catalyst bed at 100°C to start the reaction. A final difficulty is that these previous approaches all require the reactor to be completely shut off from the outside air to keep the catalyst active.

In the present study, we investigated a method to rapidly initiate oxidative decomposition of ammonia over a Ce_0.5_Zr_0.5_O_2_ supported, non-precious metal catalyst starting from room temperature. Within this group of catalysts, Co/Ce_0.5_Zr_0.5_O_2_ has been found to initiate the reaction most rapidly upon microwave irradiation. Currently, using microwave irradiation to trigger chemical reactions is attracting attention not only at the research level^21^^,^^22^ but also at the industrial level, and a national project is underway in Japan to realize energy-saving chemical processes, such as the cracking of naphtha, that utilize microwave irradiation.^23^ To use microwave irradiation to trigger a reaction, the catalyst bed is heated by irradiation at 2.45GHz ±50 MHz (the frequency currently used in household microwave ovens in Japan), which heats the catalyst bed directly and locally.^24^^,^^25^^,^^26^^,^^27^^,^^28^ This approach requires minimal energy input and allows for more rapid heating of the catalyst bed compared to conventional heating from the outside with an electric furnace. Importantly, the rapid heating allowed the use of inexpensive, non-precious metals such as cobalt, it eliminated the need for catalyst pre-treatment, and it eliminated the need for a reactor that is completely shut off from the outside air. Ce_0.5_Zr_0.5_O_2_ was used because it is reported to facilitate excellent ignition of ammonia combustion^19^ due to the contribution of mobile oxygen when used as catalyst support.^29^^,^^30^^,^^31^^,^^32^

## Results and discussion

### Triggering behavior of Co/Ce_0.5_Zr_0.5_O_2_ with microwave irradiation

**Figure 1:**
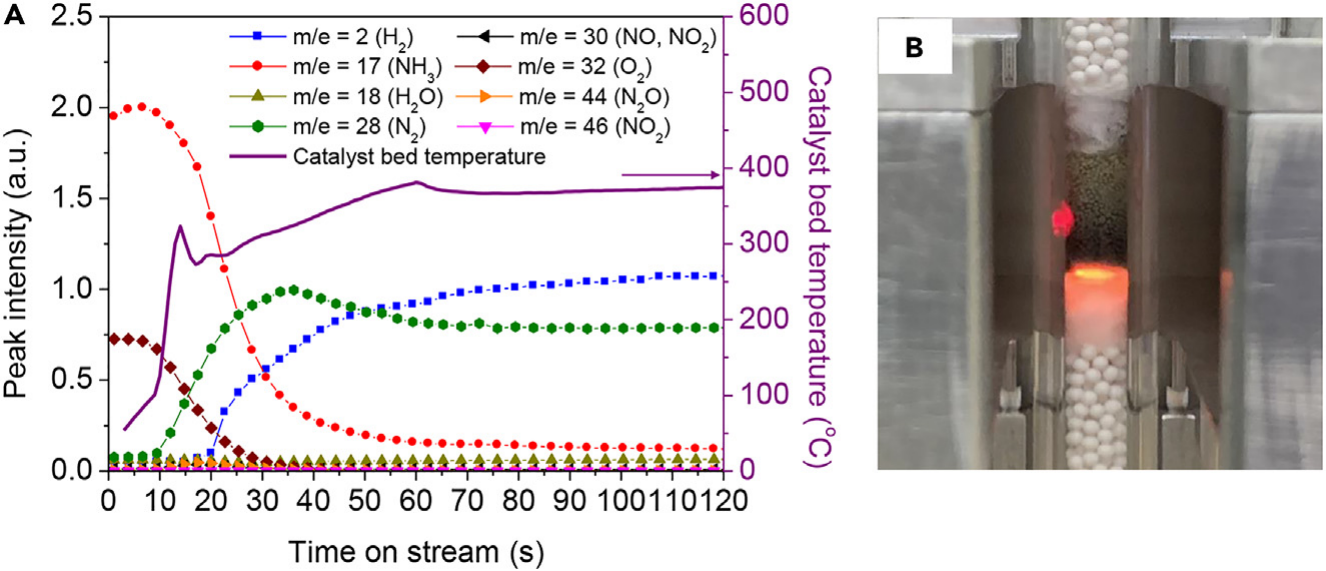
Triggering test over 20 wt % Co/Ce_0.5_Zr_0.5_O_2_ with microwave irradiation (A) Time course of mass-spectrometer intensities of the outlet gas and catalyst bed temperature. (B) Photograph of the catalyst bed after triggering oxidative decomposition of ammonia. Reaction conditions: NH_3_/O_2_/He = 150/37.5/20.8 (mL min^−1^); SV = 31.2 L h^−1^ g^−1^; no catalyst pre-treatment; initial temperature: ambient temperature; non-adiabatic conditions; microwave frequency: 2.45 GHz; output power: 10 W; irradiation time: 60 s (see also Figures S1, S2 and S12).

First, we conducted a triggering test using 20 wt % Co/Ce_0.5_Zr_0.5_O_2_ without any pre-treatment for 30 min. Figure 1A shows the time courses of the mass-spectrometer peak intensity and catalyst bed temperature when the catalyst was subjected to microwave irradiation for 60 s at 10 W (microwave frequency: 2.45GHz ±50 MHz) (See Figures S1 and S2 for the experimental setup).

At the start of irradiation, NH_3_ and O_2_, supplied as the feed gas, were detected, and the catalyst bed temperature, measured at the vertical outer surface of the catalyst bed with an infrared thermograph, increased rapidly at a rate of 500 °C min^−1^. The rise in the catalyst bed temperature further accelerated at 9 s to a rate of 2400 °C min^−1^ concomitant with the start of N_2_ formation and decreases in the amounts of NH_3_ and O_2_ produced, indicating the start of NH_3_ combustion (Equation 3). At 14 s, the catalyst bed temperature decreased once but again began to increase from 17 s, albeit at a slower rate than before. Formation of H_2_ was observed from 15 s, indicating that endothermic NH_3_ decomposition had begun (Equation 1). Thus, oxidative decomposition of ammonia was rapidly triggered rapidly from room temperature by using localized heat generated from inside the catalyst bed by microwave irradiation.

A slight drop in catalyst bed temperature was observed at 60 s, which was attributed to the suspension of the microwave irradiation. The higher temperature at the inlet than at the outlet of the catalyst bed (Figure 1B) indicated that ammonia combustion had occurred (Equation 3), followed by ammonia decomposition (Equation 1) on the catalyst bed. The temperature of the catalyst bed and formation of the gasses had stabilized by 120 s, indicating that the exothermic and endothermic reactions, including heat loss, were almost balanced.^18^ After 30 min of reaction, all of the O_2_ had been consumed, the ammonia conversion was 98 ± 1.2%, and the yield of H_2_ was 63 ± 0.7% (Table 1). The rate of formation of H_2_ was 21 ± 0.2 L h^−1^ g^−1^. Some NO, NO_2_, and N_2_O formation was observed, but only at the beginning of the reaction (from 10 to 30 s). When the triggering test was conducted with 20 wt % Co/γ-Al_2_O_3_, neither ammonia combustion nor oxidative decomposition was initiated, even with microwave irradiation at 10 W for 300 s (Figure S3). Indeed, the temperature in the bed reached only 110°C after 300 s. Together, these results indicate the importance of the choice of support material.

**Table 1:** Ammonia and oxygen conversions and hydrogen yields obtained during the triggering tests over various metal catalysts, minimum microwave irradiation (MW) output power required for triggering, and specific surface areas of the fresh catalysts

Catalyst	Specific surface area (m^2^ g^−1^)	MW output power required for triggering with 5 min (W)	NH_3_ conversion (%)	O_2_ conversion (%)	H_2_ yield (%)
20 wt % V/Ce_0.5_Zr_0.5_O_2_	22.1	10	69	100	35
20 wt % Cr/Ce_0.5_Zr_0.5_O_2_	38.6	30	60	100	26
20 wt % Mn/Ce_0.5_Zr_0.5_O_2_	29.2	10	47	100	13
20 wt % Fe/Ce_0.5_Zr_0.5_O_2_	29.9	100	80	100	46
20 wt % Co/Ce_0.5_Zr_0.5_O_2_	26.6	10	98 ± 1.2^b^	100	63 ± 0.7^b^
		External heating by electric furnace^a^	90	100	56
		10 (Pre-treatment: H_2_, 500°C, 1 h)	88	100	57
20 wt % Ni/Ce_0.5_Zr_0.5_O_2_	33.1	10	96 ± 2.1^b^	100	61 ± 1.9^b^
20 wt % Cu/Ce_0.5_Zr_0.5_O_2_	27.8	10	57	100	23
20 wt % Zn/Ce_0.5_Zr_0.5_O_2_	26.0	50	33	100	0
20 wt % Mo/Ce_0.5_Zr_0.5_O_2_	25.9	50	76	100	41
20 wt % W/Ce_0.5_Zr_0.5_O_2_	27.7	100	54	100	19
Ce_0.5_Zr_0.5_O_2_	43.3	100	33	100	2
20 wt % Co/γ-Al_2_O_3_	122.4	30	77	100	43
1 wt % Ru/Ce_0.5_Zr_0.5_O_2_	52.0	30	94	100	62

a The Experimental setup is shown in Figure S4.

b Data are represented as mean ± SEM of at least three independent experiments.

### Comparison of catalytic behavior triggered by microwave irradiation or conventional heating

Next, we examined the difference in the catalytic behavior of a 20 wt % Co/Ce_0.5_Zr_0.5_O_2_ catalyst after triggering by heating from ambient temperature with microwave irradiation or a conventional electric furnace. In the test using the electric furnace, three thermocouples were inserted into the catalyst bed to measure the temperature at the horizontal center of the catalyst bed (see Figure S4 for the experimental setup). The time dependence of the temperatures of each thermocouple is shown in Figure S5. At 11 min after the start of heating, the temperature rapidly increased, indicating that oxidative decomposition of ammonia had been triggered. The temperature needed to trigger the reaction was 198°C, although the rate of the temperature increase was set at 20°C/min, indicating that the catalyst could not be heated at the set rate (20°C/min) with electric heating. After confirming that the reaction had been triggered with the drastic rise in the catalyst bed temperature, the heating of the furnace was stopped. At 15 min, the temperatures in the three parts of the catalyst bed reached a steady state, with the temperature at the inlet part of the catalyst bed being much higher than that at the outlet part of the catalyst bed, indicating that ammonia combustion at the inlet and ammonia decomposition at the outlet was occurring. Here, note that different steady state temperatures were recorded in the tests using the electric heater and microwave irradiation. When the electric heater was used, the temperature in the middle part of the catalyst, as measured by thermocouple, stabilized at around 550°C; whereas, when microwave irradiation was used, the temperature at the outer surface of the vertical center of the catalyst bed, as measured by infrared thermograph, stabilized at around 400°C. Thus, the temperature at the horizontal center of the catalyst was much higher than that of the catalyst bed measured at the vertical outer surface of the catalyst bed by infrared thermograph. The NH_3_ and O_2_ conversions and H_2_ yields over Co/Ce_0.5_Zr_0.5_O_2_ catalyst after switching off the electric heater are shown in Table 1. The NH_3_ conversion and H_2_ yield in the triggering test with microwave irradiation were 7–10% higher than those obtained with the electric heater. The difference in NH_3_ conversion might be ascribed to the large differences in the rates of temperature increase with the different heating methods (microwave irradiation: >500°C/min; electric furnace: <20°C/min).

X-ray diffraction (XRD) patterns were recorded after 30 min of triggering the oxidative decomposition of ammonia with the two heating types, with the reactions being immediately halted by replacing the feed gas with helium. In the XRD pattern recorded after the reaction triggered with the electric furnace, the production of CoO and Co^0^ was observed; whereas in the XRD pattern recorded after the reaction triggered with microwave irradiation only the production of Co^0^ was observed (Figure S6). Thus, rapid heating of the catalyst from ambient temperature to the triggering temperature by means of microwave irradiation avoided degradation of the catalyst due to Co oxidation.

### Influence of metal species on the catalytic behavior of Ce_0.5_Zr_0.5_O_2_-supported metal catalysts heated by microwave irradiation

It is important to trigger the oxidative ammonia decomposition within a short time of starting microwave irradiation to minimize input and maximize NH_3_ conversion, O_2_ conversion, and H_2_ yield. Therefore, we investigated the effects of different supported non-precious metal species on the triggering behavior and activity of the catalysts once steady state had been reached at 30 min from the start of heating by microwave irradiation.

**Figure 2:**
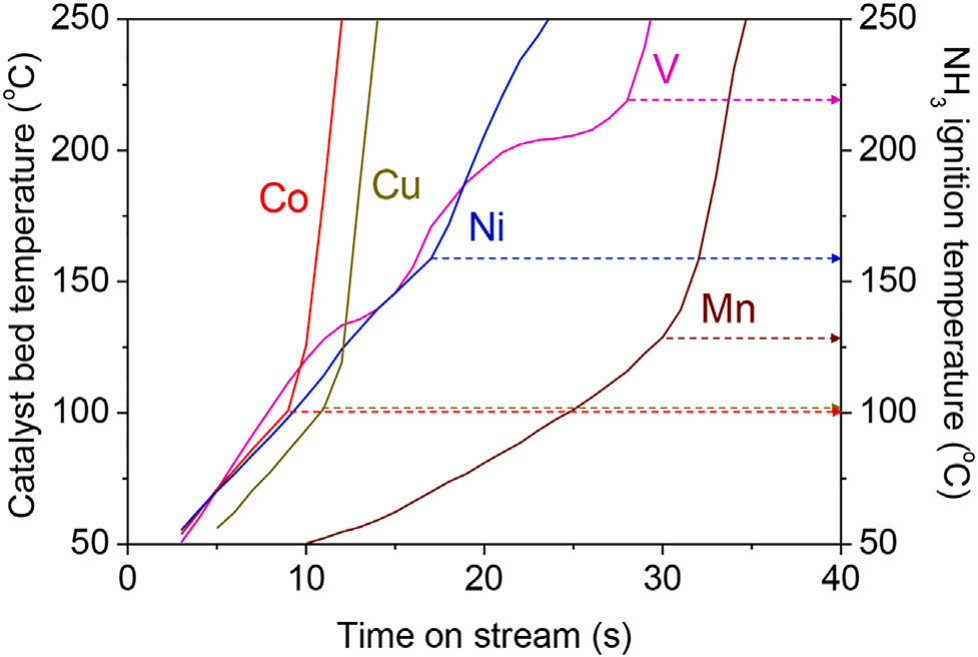
Time course of catalyst bed temperature during the triggering tests using different Ce_0.5_Zr_0.5_O_2_-supported metal catalysts and microwave irradiation, for catalysts with which the reaction was triggered within 60 s from the start of heating The catalytic auto-ignition temperature of each catalyst is also shown by the arrow on the right axis in the figure. Reaction conditions: NH_3_/O_2_/He = 150/37.5/20.8 (mL min^−1^); SV = 31.2 L h^−1^ g^−1^; no catalyst pre-treatment; initial temperature: ambient temperature; non-adiabatic conditions; microwave frequency: 2.45 GHz; output power: 10 W; irradiation time: 60 s.

Figure 2 shows the time dependence of the catalyst bed temperature during the triggering tests. In these tests, microwave irradiation was first performed at an output power of 10 W, and if the reaction was triggered within 60 s, irradiation was stopped at 60 s. For the catalysts containing Co, Cu, Ni, V, and Mn, oxidative decomposition of ammonia was triggered within 60 s. The Co and Cu catalysts in particular were heated rapidly, and the ammonia auto-ignition temperatures were lower than those of the other catalysts. In contrast, Ni and V catalysts were rapidly heated, but the ammonia auto-ignition temperature was higher than 150°C. The different heating rates of the different catalysts indicated that the loaded metal species played a crucial role in altering the susceptibility of the catalyst bed to heating by microwave irradiation. On the other hands, bare Ce_0.5_Zr_0.5_O_2_ was not heated to even 50°C within 60 s, the lowest temperature that our infrared thermograph could measure, emphasizing importance of the loaded metal species for increasing the heating rate.

**Figure 3:**
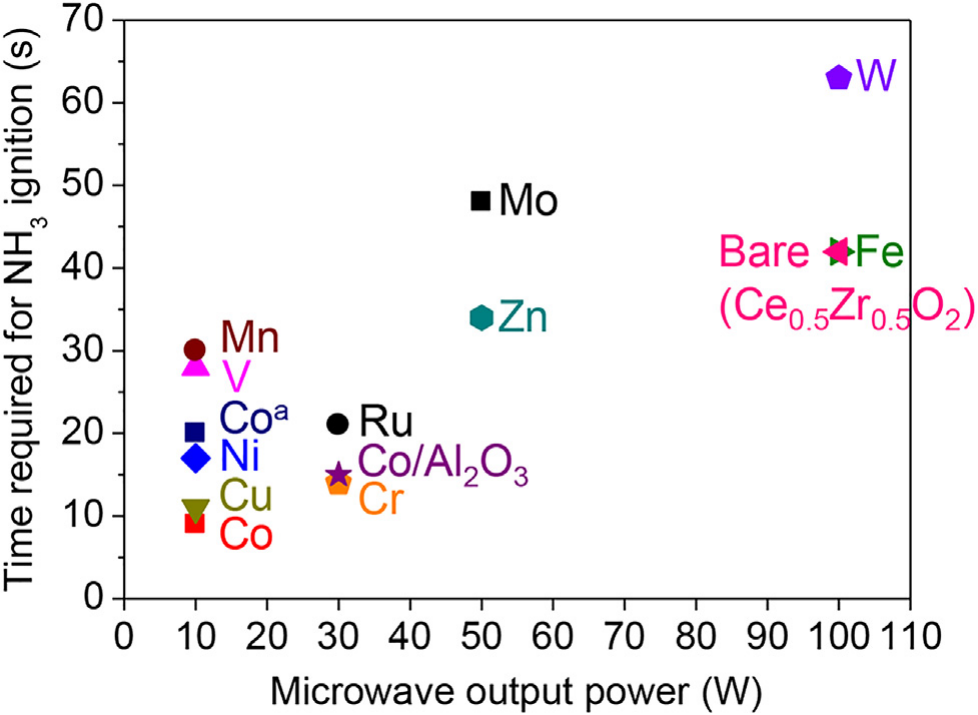
Minimum microwave output power and irradiation time required to trigger oxidative ammonia decomposition over various Ce_0.5_Zr_0.5_O_2_-supported non-noble metal catalysts, bare Ce_0.5_Zr_0.5_O_2_, and Co/Al_2_O_3_ Reaction conditions: NH_3_/O_2_/He = 150/37.5/20.8 (mL min^−1^); SV = 31.2 L h^−1^ g^−1^; no catalyst pre-treatment; initial temperature: ambient temperature; non-adiabatic conditions; microwave frequency: 2.45 GHz; microwave output power: 10, 30, 50, or 100 W ^a^Pre-treatment: H_2_, 500°C, 1 h.

Figure 3 shows the microwave output power and time taken to trigger NH_3_ combustion over the various loaded catalysts. When the reaction was not triggered even after 5 min of microwave irradiation at 10 W, the output power was increased, step-by-step, to 30, 50, and 100 W, with 5 min irradiation time for each step starting with the catalyst at ambient temperature. With microwave irradiation at 10 W, oxidative decomposition of ammonia was triggered over the Co, Cu, Ni, V, and Mn catalysts only. For the Cr and Co/γ-Al_2_O_3_ catalysts, irradiation at 30 W was required; for the Zn and Mo catalysts, irradiation at 50 W was required; and for the Fe and W catalysts and bare Ce_0.5_Zr_0.5_O_2_, irradiation at 100 W was required. We also tested 1 wt % Ru/Ce_0.5_Zr_0.5_O_2_, which we previously used successfully to trigger oxidative ammonia decomposition from ambient temperature by using the heat generated through oxidation of the reduced catalyst.^19^ For this catalyst, irradiation at 30 W was required because of the low loading of Ru.

To investigate the effect of pre-treatment, we pre-reduced Co/Ce_0.5_Zr_0.5_O_2_ at 500°C in pure H_2_ for 1 h before the triggering test. In this case, the reaction was triggered, but only by irradiation at 10 W for 20 s. After pre-reduction, the catalyst was purged with He for 30 min at 500°C, cooled to ambient temperature, and exposed to O_2_/He (1/4.6 v/v). The catalyst was then used in a triggering test using a mixture of NH_3_, O_2_, and He. During the O_2_ and He treatment, the Ce_0.5_Zr_0.5_O_2-*x*_ and Co^0^ in the reduced catalyst were oxidized and heated to 332°C, which induced oxidation of metallic Co to Co_3_O_4_, sintering of Co_3_O_4_, and an increase of the triggering temperature from 101°C to 176°C.

Overall, the Co/Ce_0.5_Zr_0.5_O_2_ catalyst without pre-treatment exhibited the best catalytic performance, being able to trigger the reaction within 9 s and at only 101 °C at a minimum output power of 10 W.

**Figure 4:**
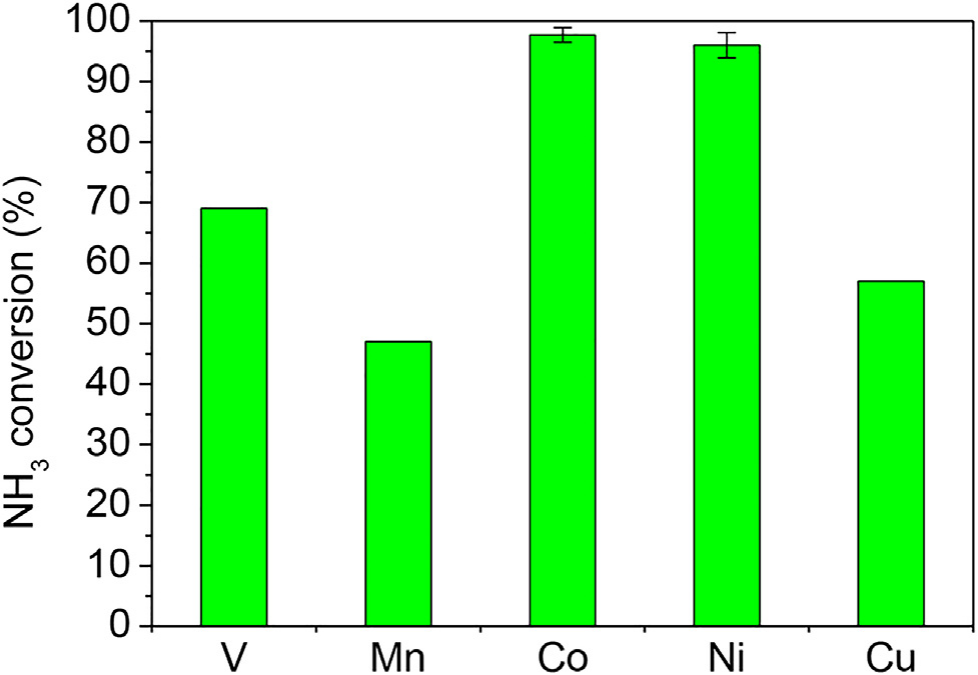
Ammonia conversions after 30 min from the start of oxidative decomposition of ammonia over Ce_0.5_Zr_0.5_O_2_-supported metal catalysts for which the reaction was triggered with microwave irradiation at 10 W Reaction conditions: NH_3_/O_2_/He = 150/37.5/20.8 (mL min^−1^); SV = 31.2 L h^−1^ g^−1^; no catalyst pre-treatment; initial temperature: ambient temperature; non-adiabatic conditions; microwave frequency: 2.45 GHz; output power: 10 W; irradiation time: 60 s. Data for Co and Ni catalysts are represented as mean ± SEM of at least three independent experiments.

We then evaluated ammonia conversions at 30 min from the start of the oxidative decomposition of ammonia using the catalysts that triggered the reaction when irradiated at 10 W (i.e., Co, Cu, Ni, V, and Mn) (Figure 4; Table 1). The Co and Ni catalysts afforded ammonia conversions greater than 95% and H_2_ yields greater than 60%, whereas the V, Mn, and Cu catalysts afforded ammonia conversions of less than 70%. Note that the ammonia conversion of bare Ce_0.5_Zr_0.5_O_2_ was 33% and the hydrogen conversion was 2%, indicating that metallic elements are essential for hydrogen production. O_2_ conversions were 100% for all of the catalysts, indicating that ammonia combustion progressed until all of the oxygen was consumed. For the other catalysts containing non-precious metals, the Fe and Mo catalysts showed ammonia conversions greater than 75%. H_2_ pre-treatment of the Co catalyst resulted in lower ammonia conversion than Co catalyst without pre-treatment because of sintering of Co_3_O_4_ (Table 1). The Ru catalyst afforded a high ammonia conversion of 94%, even with a Ru loading of only 1 wt %.

**Figure 5:**
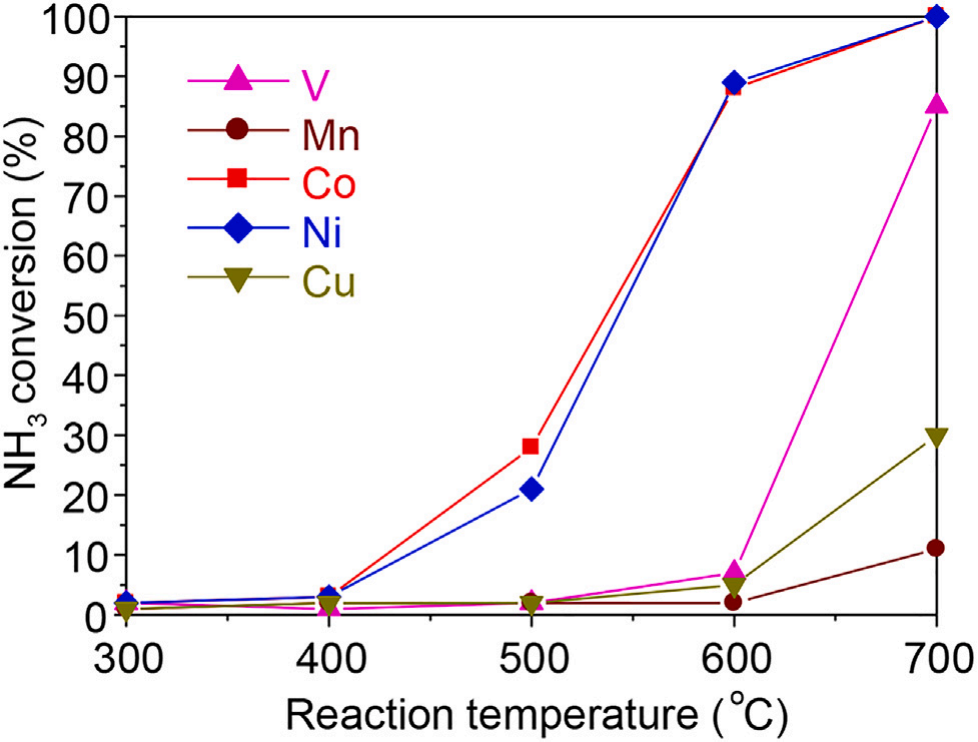
Temperature dependence of ammonia conversions during the ammonia decomposition reaction over 20 wt % Co, Cu, Ni, V, or Mn/Ce_0.5_Zr_0.5_O_2_ catalyst Reaction conditions: NH_3_/He = 15/5 (mL min^−1^); SV = 12.0 L h^−1^ g^−1^; pre-treatment: H_2_, 700°C, 1 h; catalyst amount: 100 mg.

Next, we evaluated the ammonia decomposition (Equation 1) activity of the catalysts as a function of reaction temperature with an electric furnace (Figure 5). The reaction started at 500°C for the Co and Ni catalysts and at 600°C for the V, Cu, and Mn catalysts. NH_3_ conversions of the catalysts at 700°C decreased in the order Co = Ni > V > Cu > Mn. This order is in agreement with the order of NH_3_ conversion triggered from ambient temperature with microwave irradiation (Figure 4), revealing that NH_3_ conversion by oxidative decomposition is determined by the ammonia decomposition ability of the catalyst, which proceeds by using the heat produced by ammonia combustion.

**Figure 6:**
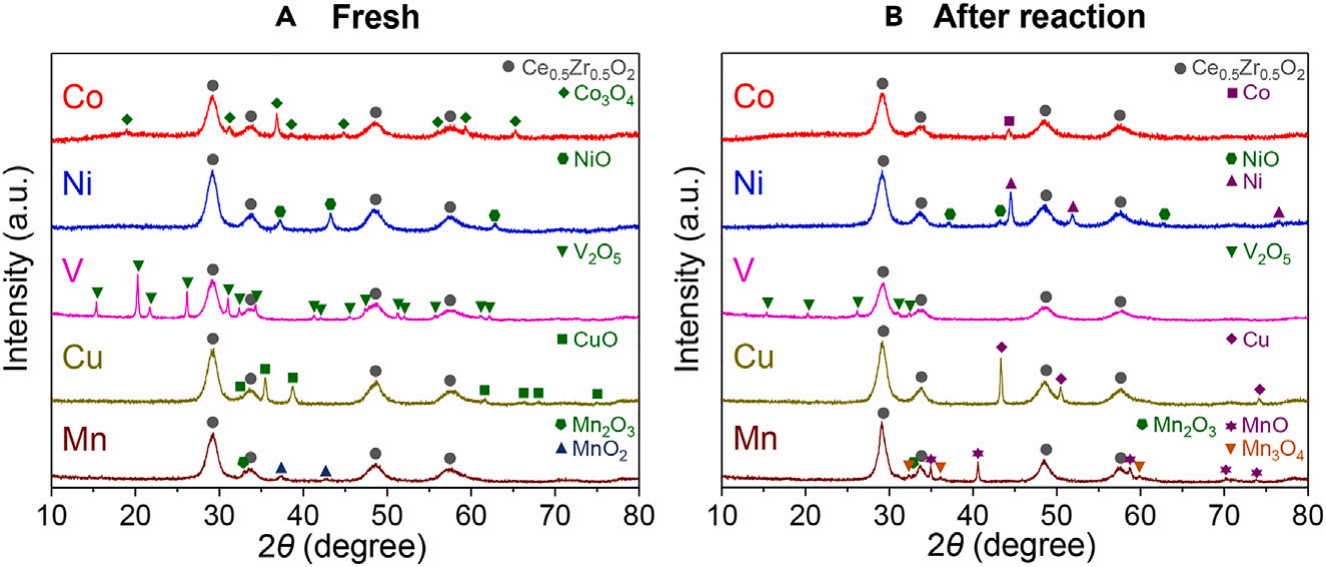
X-ray diffraction patterns of 20 wt % Co, Ni, V, Cu, or Mn/Ce_0.5_Zr_0.5_O_2_ catalyst (A) before and (B) after the triggering test with microwave irradiation at 10 W. In (B), the catalysts were collected after halting the reaction after 30 min of the reaction by replacing the feed gas mixture with He.

To understand more about the structural changes the catalysts underwent during the triggering tests with microwave irradiation, the reaction was halted after 30 min of the reaction by replacing the feed gas with He, the reactor was quenched to ambient temperature, and XRD patterns of the catalysts were recorded and compared with those of the fresh catalysts (Figure 6). For the fresh catalysts, production of the oxides Co_3_O_4_, NiO, V_2_O_5_, CuO, MnO_2_, and Mn_2_O_3_ was observed, which is attributed to the catalysts being calcined at 450°C during their preparation (Figure 6A). However, after the triggering tests, the oxides were reduced, and Co^0^, Ni^0^, Cu^0^, and MnO were observed (Figure 6B). These results indicate that the oxidative decomposition of ammonia is catalyzed by metals in reduced states.

### Cycling and durability tests

**Figure 7:**
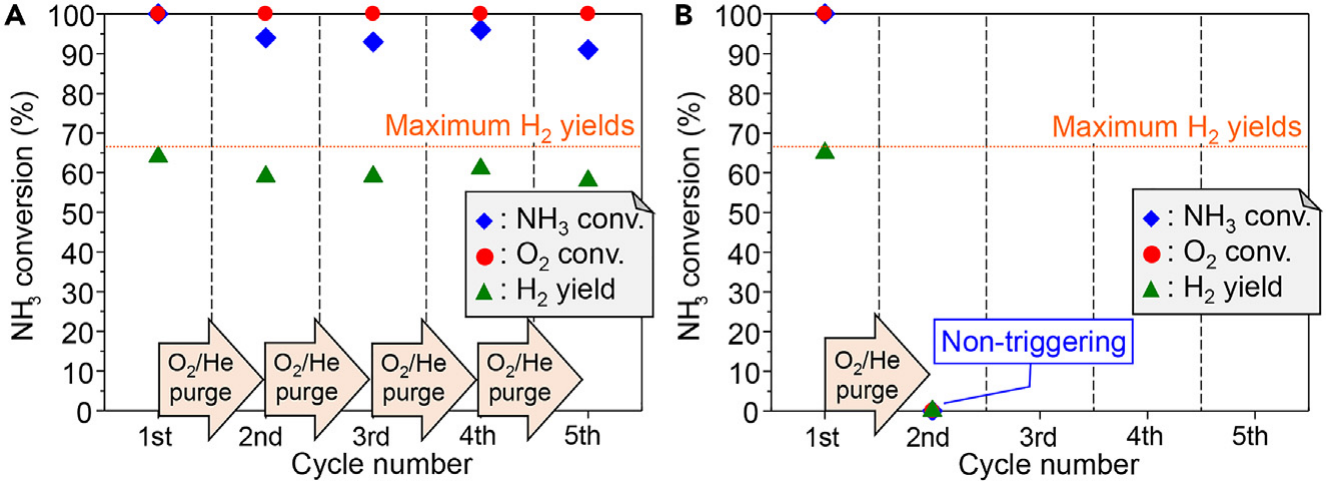
Catalytic activity during the cycling test (A) 20 wt % Co/Ce_0.5_Zr_0.5_O_2_ and (B) 20 wt % Ni/Ce_0.5_Zr_0.5_O_2_. Reaction conditions: NH_3_/O_2_/He = 150/37.5/20.8 (mL min^−1^); SV = 31.2 L h^−1^ g^−1^; no catalyst pre-treatment; initial temperature: ambient temperature; non-adiabatic conditions; microwave frequency: 2.45 GHz; microwave output power: 10 W; irradiation time: 1–5 min (when the reaction was not triggered within 1 min, an addition 1 min of irradiation time was added up to a maximum of 5 min); purge gas: O_2_/He (see also Figure S7).

Because the Co and Ni catalysts showed good triggering behavior with microwave irradiation and high NH_3_ conversions after 30 min, cycling tests were performed for these catalysts (Figure 7). In this test, the first cycle was performed with the procedure already shown. Then, at 30 min after the start of the reaction, the reaction was terminated by stopping the NH_3_ supply, and the catalyst was cooled to ambient temperature in an O_2_/He mixture. Once at ambient temperature, NH_3_ gas was added to the O_2_/He flow (NH_3_:O_2_:He = 150:37.5:20.8 [mL min^−1^]) to remove the effect of heat produced by ammonia adsorption.^5^ Then, a second triggering was attempted, again by using microwave irradiation with 10 W for 60 s. This feed-and-purge cycle was repeated for a total of five cycles (see Figure S7 for procedure).

Co/Ce_0.5_Zr_0.5_O_2_ catalyst afforded complete consumption of O_2_, an NH_3_ conversion higher than 90% with only a slight decrease with each cycle, and an H_2_ yield near the maximum value during all five cycles (Figure 7A). These results indicated that the Co catalyst was able to repeatedly trigger the oxidative decomposition of ammonia and maintain high activity up to the fifth cycle. High-angle annular dark-field scanning transmission electron microscopy and energy dispersive X-ray analyses before and after the five cycles of the recycling test showed increase in the particle size of Co species and Ce_0.5_Zr_0.5_O_2_ during the test, indicating that the slight decrease in activity was due to sintering of the catalyst (Figures S8 and S9). In contrast, the Ni catalyst could not trigger the reaction repeatedly over multiple cycles, as no hydrogen was produced even after 5 min of microwave irradiation at 10 W in the second cycle (Figure 7B). These contrary findings can be explained as follows: Ni^0^ and Co^0^ catalyzed the oxidative decomposition of ammonia. When the reaction was stopped by replacing the feed gas with He, only Co^0^ was observed in the Co catalyst (Figure S10). In contrast, for the Ni catalyst, NiO as well as Ni^0^ was observed, indicating that some of the Ni was oxidized and degraded (Figure S11). Furthermore, XRD patterns were recorded for both catalysts after cooling down to ambient temperature in an O_2_/He mixture before the second cycle (Figures S10 and S11). In the case of the Ni catalyst, the crystallite size of NiO was estimated to be 55 nm based on the peak of the (200) plane, indicating that the NiO was strongly sintered. However, the crystallite size of CoO was estimated to be 37 nm using the same plane, indicating that sintering of CoO was minor and that the Co catalyst remained relatively stable during the cycling test.

**Figure 8:**
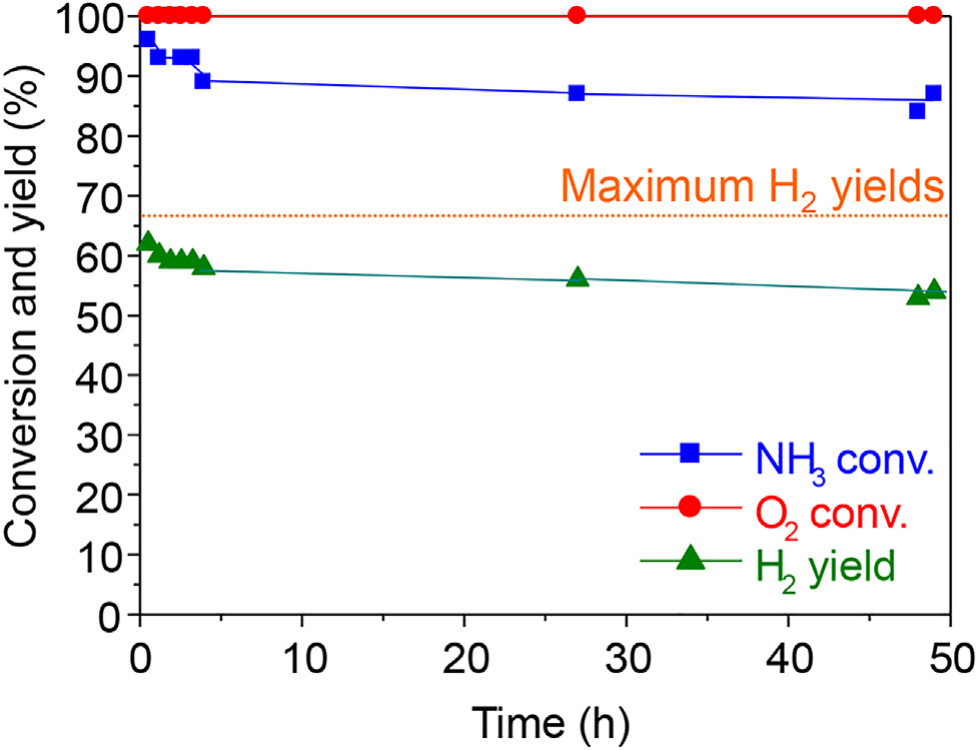
Catalytic activity of 20 wt % Co/Ce_0.5_Zr_0.5_O_2_ in a long-term test for 48 h Reaction conditions: NH_3_/O_2_/He = 150/37.5/20.8 (mL min^−1^); SV = 31.2 L h^−1^ g^−1^; no catalyst pre-treatment; initial temperature: ambient temperature; non-adiabatic conditions; microwave frequency: 2.45 GHz; output power: 10 W; irradiation time: 60 s.

We also conducted a durability test. Co/Ce_0.5_Zr_0.5_O_2_ showed an initial decrease in activity probably due to sintering as during the cycle tests, but the activity then stabilized out to almost 48 h without the need for heating (Figure 8). Based on both the recycling and durability tests, we concluded that Co/Ce_0.5_Zr_0.5_O_2_ was a suitable catalyst for the oxidative decomposition of ammonia triggered by microwave irradiation starting from ambient temperature.

### Conclusions

Ammonia is a promising carbon-free fuel for use in power plants. However, since ammonia has low flammability and is difficult to ignite, it must be burned with hydrogen as a combustion improver. Therefore, an efficient process for the production of H_2_ from ammonia using non-precious metal catalysts is needed. Here, we found that microwave irradiation almost instantly triggered the oxidative decomposition of ammonia to produce hydrogen at a high rate over Ce_0.5_Zr_0.5_O_2_-supported non-precious metal catalysts. The localized heat generated by microwave irradiation rapidly raised the catalyst bed temperature to the auto-ignition temperature of ammonia, and then hydrogen production by oxidative decomposition of ammonia was initiated. Over Co/Ce_0.5_Zr_0.5_O_2_, oxidative ammonia decomposition could be repeatedly triggered from room temperature by microwave irradiation because of the catalyst’s high thermal stability. Negligible amounts of NO_*x*_ were emitted during start-up. In future research, we intend to build on these findings and develop catalysts that heat more rapidly and need less microwave irradiation. Our present discovery is expected to contribute to the development of efficient ammonia-fueled power generation systems and a carbon-neutral society.

### Limitations of the study

The most important limitation of the present study is that the reactions were performed by using hundred milligrams of catalysts. Since oxidative decomposition is an exothermic reaction, scale-up testing will be needed before an industrial process can be established.

## STAR★Methods

### Key resources table

**Table:** 

REAGENT or RESOURCE	SOURCE	IDENTIFIER
**Chemicals, peptides, and recombinant proteins**		

ZrO(NO_3_)_2_·2H_2_O	KANTO CHEMICAL CO., INC.	CAS 20213-65-4
Ce(NO_3_)_3_·6H_2_O	KANTO CHEMICAL CO., INC.	CAS 10294-41-4
28% Ammonia solution	FUJIFILM Wako Pure Chemical Corp.	CAS 1336-21-6
NH_4_VO_3_	FUJIFILM Wako Pure Chemical Corp.	CAS 7803-55-6
(CH_3_COO)_3_Cr	FUJIFILM Wako Pure Chemical Corp.	CAS 1066-30-4
Mn(NO_3_)_2_·6H_2_O	FUJIFILM Wako Pure Chemical Corp.	CAS 17141-63-8
Fe(NO_3_)_3_·9H_2_O	FUJIFILM Wako Pure Chemical Corp.	CAS 7782-61-8
Co(NO_3_)_2_·6H_2_O	FUJIFILM Wako Pure Chemical Corp.	CAS 10026-22-9
Ni(NO_3_)_2_·6H_2_O	FUJIFILM Wako Pure Chemical Corp.	CAS 13478-00-7
Cu(NO_3_)_2_·3H_2_O	FUJIFILM Wako Pure Chemical Corp.	CAS 10031-43-3
Zn(NO_3_)_2_·6H_2_O	FUJIFILM Wako Pure Chemical Corp.	CAS 10196-18-6
(NH_4_)_6_Mo_7_O_24_·4H_2_O	FUJIFILM Wako Pure Chemical Corp.	CAS 12054-85-2
(NH_4_)_10_W_12_O_41_·5H_2_O	FUJIFILM Wako Pure Chemical Corp.	CAS 1311-93-9
Ru_3_(CO)_12_	Tanaka Kikinzoku Kogyo K.K.	CAS 15243-33-1
9 mol/L(18N)-Sulfuric acid	KISHIDA CHEMICAL CO., LTD.	CAS 7664-93-9

**Software and algorithms**		

MicroWave version 1.0.8.0	Ryowa Electronics Co., Ltd.	N/A
BELMass version 1.3.0.6	MicrotracBEL Corp.	N/A
BELSORP-miniX version 1.1.3.0	MicrotracBEL Corp.	N/A
PDXL2 version 2.8.4.0	Rigaku Corp.	https://www.rigaku.com/support/software
Origin version 6.0	OriginLab Corp.	https://www.originlab.com/index.aspx?go=Support&pid=450

**Other**		

Microwave Reactor MR-2G-100HPAAC	Ryowa Electronics Co., Ltd.	http://www.ryowa-electronics.co.jp/products/mr/index.html
Infrared Thermometer TMHX-STM0050-0070E003-0445	JAPANSENSOR Corp.	https://www.japansensor.co.jp/english/
Quadrupole Mass Spectrometer BELMASS	MicrotracBEL Corp.	https://www.microtrac.com/products/selection-guides/model-list-a-z/
BET Specific Surface Area & Pore Size Analyzer BELSORP MINI X	MicrotracBEL Corp.	https://www.microtrac.com/products/gas-adsorption-measurement/surface-area-pore-size-distribution/belsorp-mini-x/
Gas Chromatograph GC-8A	Shimadzu Corp.	https://www.shimadzu.com/an/products/gas-chromatography/index.html
Packed Column Shincarbon-ST	Shinwa Chemical Industries Ltd.	https://shinwa-cpc.co.jp/en/products/gc/packed/list/
XRD SmartLab	Rigaku Corp.	https://www.rigaku.com/products/xrd/smartlab
TEM JEM-ARM200F	JEOL	https://www.jeol.com/products/scientific/tem/JEM-ARM200F_NEOARM.php

### Resource availability

#### Lead contact

Further information and requests for resources and reagents should be directed to and will be fulfilled by the lead contact, Katsutoshi Nagaoka (nagaoka.katsutoshi@material.nagoya-u.ac.jp).

#### Materials availability

This study did not generate new unique reagents.

#### Data and code availability

• All data reported in this paper will be shared by the lead contact upon request.
• This paper does not report original code.
• Any additional information required to reanalyze the data reported in this paper is available from the lead contact upon request.

### Method details

#### Catalyst preparation

Ce_0.5_Z_r0.5_O_2_ support was prepared by the co-precipitation method described in an earlier report.^33^ In short, ZrO(NO_3_)_2_·2H_2_O and Ce(NO_3_)_3_·6H_2_O (KANTO CHEMICAL CO., INC., Japan) with a molar ratio of 1:1 were dissolved in purified water, this solution was added to a 28% NH_3_ solution (FUJIFILM Wako Pure Chemical Corporation, Japan), and a precipitate was formed. The precipitate was kept in suspension by stirring overnight at 300 rpm at room temperature, retrieved by filtration, washed with pure water, and dried at 80°C overnight. The obtained powder was calcined in static air at 700°C for 5 h in a muffle furnace with the ramping rate set at 10°C/min. Then, precursors of transition metals with 20 wt % against the Ce_0.5_Zr_0.5_O_2_ support were loaded by a wet impregnation method. NH_4_VO_3_, (CH_3_COO)_3_Cr, Mn(NO_3_)_2_·6H_2_O, Fe(NO_3_)_3_·9H_2_O, Co(NO_3_)_2_·6H_2_O, Ni(NO_3_)_2_·6H_2_O, Cu(NO_3_)_2_·3H_2_O, Zn(NO_3_)_2_·6H_2_O, (NH_4_)_6_Mo_7_O_24_·4H_2_O, and (NH_4_)_10_W_12_O_41_·5H_2_O were used as the metal precursors (FUJIFILM Wako). The Ce_0.5_Zr_0.5_O_2_ support was added to an aqueous solution of the metal precursor and stirred overnight at room temperature. The suspension was dried by using a hot stirrer at 80°C and atmospheric pressure. The resulting powder was dried in an oven at 80°C overnight and then calcined in flowing dried air in a tube furnace at 450°C for 5 h (ramping rate: 5°C/min) to remove counter anions such as NO_3_^−^ from the precursors.

#### Triggering tests

The catalyst powder was pressed into a pellet using a pressure of 30 MPa, crushed, and sieved to grains with a largest dimension of 250–500 μm. Then, 400 mg of the catalyst grains were installed in a fixed bed flow system (Figure S1) by loading them into a tubular quartz reactor (i.d. 5 mm), the ends of which were plugged with quartz wool. The lower and upper internal spaces of the reactor were filled with α-Al_2_O_3_ balls with a 1-mm diameter. A semiconductor microwave irradiator (MR-2G-100HPAAC, Ryowa Electronics Co., Ltd., Japan) equipped with a high-frequency power supply was used to irradiate the catalyst. The irradiator was set to produce microwaves with a frequency of 2.45GHz ±50 MHz. The irradiator was equipped with a cylindrical TM010 cavity with a 90-mm diameter and 20-mm height that was capable of generating a standing wave with the electric field distribution in the diameter direction. The catalyst was irradiated from outside of the reactor.^34^ An infrared thermograph (TMHX-STM0050-0070E003-0445, Japansensor Corporation, Japan) was attached to the microwave irradiator to measure the average temperature at the vertical outer surface of the catalyst bed; the diameter of the measuring point was 5 mm. The experimental setup is shown in Figures S1 and S2. A quadrupole mass spectrometer (BELMASS, MicrotracBEL Corp., Japan) was attached to the outlet of the reactor to monitor the composition of the gasses which include water and NH_3_ (e.g., Figure 1A). Water and NH_3_ strongly adsorb on the column and damage gas chromatograph. Therefore, the outlet gas was passed through a cold trap to remove water and an H_2_SO_4_ (9 mol L^−1^) trap to remove NH_3_. The constant volume of gases was subsequently introduced to a gas chromatograph (GC-8A, Shimadzu Corporation, Japan) equipped with a thermal conductivity detector and Shincarbon-ST column (Shinwa Chemical Industries Ltd., Japan) by using a 6-way valve connected to the sample loop (0.5 mL). Conversions and yields were calculated as follows using Helium as an internal standard.

(Equation 4) $${NH}_{3}conversion\;\left(\%\right)=\frac{\left(\frac{C_{N_{2}-out-dry}}{C_{He-out-dry}}\times F_{He-in}\right)\times 2}{F_{{NH}_{3}-in}}\times 100$$

(Equation 5) $$O_{2}\;conversion\;\left(\%\right)=\frac{F_{O_{2}-in}-\left(\frac{C_{O_{2}-out-dry}}{C_{He-out-dry}}\times F_{He-in}\right)}{F_{O_{2}-in}}\times 100$$

(Equation 6) $$H_{2}\;yield\;\left(\%\right)=\frac{\left(\frac{C_{H_{2}-out-dry}}{C_{He-out-dry}}\times F_{He-in}\right)\times 2}{F_{{NH}_{3}-in}\times 3}\times 100$$

where *C*_*out-dry*_ is the composition of effluent gases after passing through the cold trap to remove water and the H_2_SO_4_ trap to remove NH_3_; *F*_*in*_ and *F*_*out*_ are the molar flow rate of the gas species in the inlet gas and the effluent gas, respectively. Details of calculation methods, equations, and nomenclatures are described in SI (Equations S1–S10).

The typical procedure used for the triggering tests is shown in Figure S12. Reactant mixture (NH_3_/O_2_/He = 150/37.5/20.8 (mL min^−1^)) with a space velocity of 31.3 L h^−1^ g^−1^ was supplied to the catalyst bed at ambient temperature without any pre-treatment for the catalyst. The catalyst was then heated from ambient temperature with microwave irradiation at 10 W (microwave frequency: 2.45GHz ±50 MHz). Usually, the irradiation was stopped after 60 s. However, when the reaction was not triggered after 5 min of irradiation at 10 W, the output power was increased step-by-step to 30, 50, and 100 W.

For the recycling test, after 30 min of triggering the reaction in the first cycle, the reaction was halted by stopping the NH_3_ flow. Then the reactor was quenched to ambient temperature. Once at ambient temperature, NH_3_ (150 mL min^−1^) was added to the O_2_ and He gas mixture and it was observed that the catalyst bed temperature was increase slightly by the adsorption heat of NH_3_ and again cooled to ambient temperature. Then a second cycle was started, and the catalyst was again heated with microwave irradiation at 10 W for 60 s. This heat–reaction–cool process was repeated a total of five times (Figure S7).

#### Characterization

The specific surface areas of the catalysts were estimated after vacuum treatment at 300°C by using the Brunauer–Emmett–Teller method using a BELSOPRP MINI X instrument (MicrotracBEL). XRD analysis was performed with a SmartLab X-ray diffractometer (Rigaku Corporation, Japan) equipped with a Cu Kα radiation source. PDXL2 software version 2.8.4.0 (Rigaku) and the ICDD (International Center for Diffraction Data), COD (Crystallography Open Database),^35^ and AtomWork^36^ databases were used to analyze the XRD patterns.

High-angle annular dark-field scanning transmission electron microscopy images and energy dispersive X-ray elemental maps were obtained with a JEM-ARM200F electron microscope (JEOL, Japan) operated at 200 kV. Samples were dispersed in ethanol under ambient conditions, and the dispersion was dropped onto a carbon-coated copper grid and then dried under a vacuum at ambient temperature for 24 h.

### Quantification and statistical analysis

Statistical analysis of data was performed using Origin (OriginLab) and Excel (Microsoft). All data are represented as mean ± SEM. See individual figure and Table legends for details regarding number of data.

### Additional resources

This work does not include any additional resources.

## Supplemental information

Document S1. Figures S1–S12 and Equations S1–S10
