# Supplementary material for: Rapid start-up of carbon-free H2 production by ammonia oxidative decomposition over Co/Ce0.5Zr0.5O2 with microwave irradiation
Source: iScience. 2024 Jul 3;27(8):110452. doi: 10.1016/j.isci.2024.110452 (PMC11300920; doi:10.1016/j.isci.2024.110452)
Supplement: Document S1. Figures S1–S12 and Equations S1–S10 [file mmc1.pdf]

iScience, Volume *27*

Supplemental information

Rapid start-up of carbon-free H_2 production by ammonia oxidative decomposition over $Co/Ce_{0.5}Zr_{0.5}O_2$ with microwave irradiation

Takahiro Matsunaga, Sachika Hayashi, Hiroshi Yamada, Katsutoshi Sato, and Katsutoshi Nagaoka

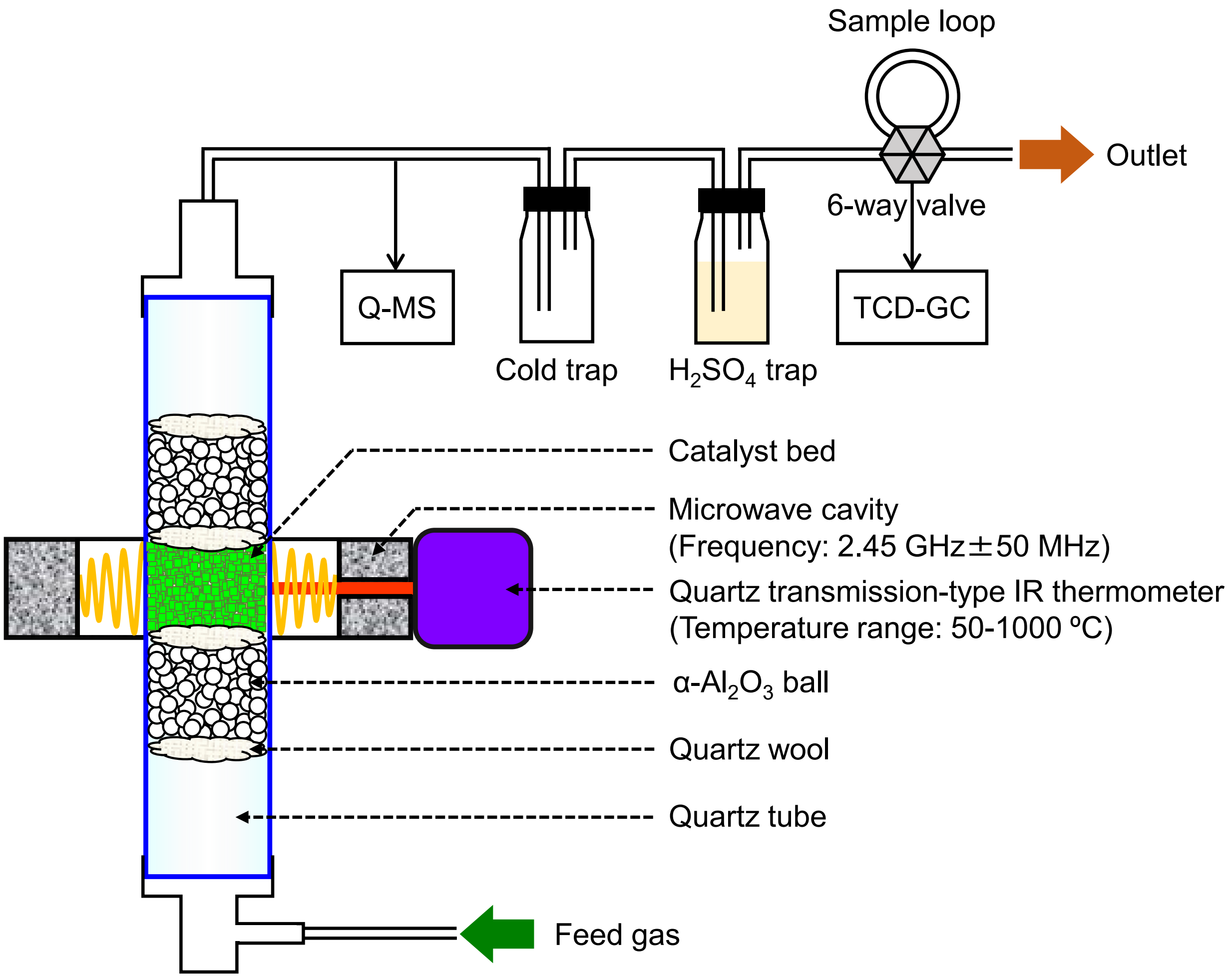

Figure S1. Schematic diagram of the non-adiabatic reactor used for the triggering tests with microwave irradiation, related to STAR Methods.

The reactor was equipped with a microwave cavity, infrared (IR) thermometer, quadrupole mass spectrometer (Q-MS), and gas chromatograph (GC) with a thermal conductivity detector (TCD).

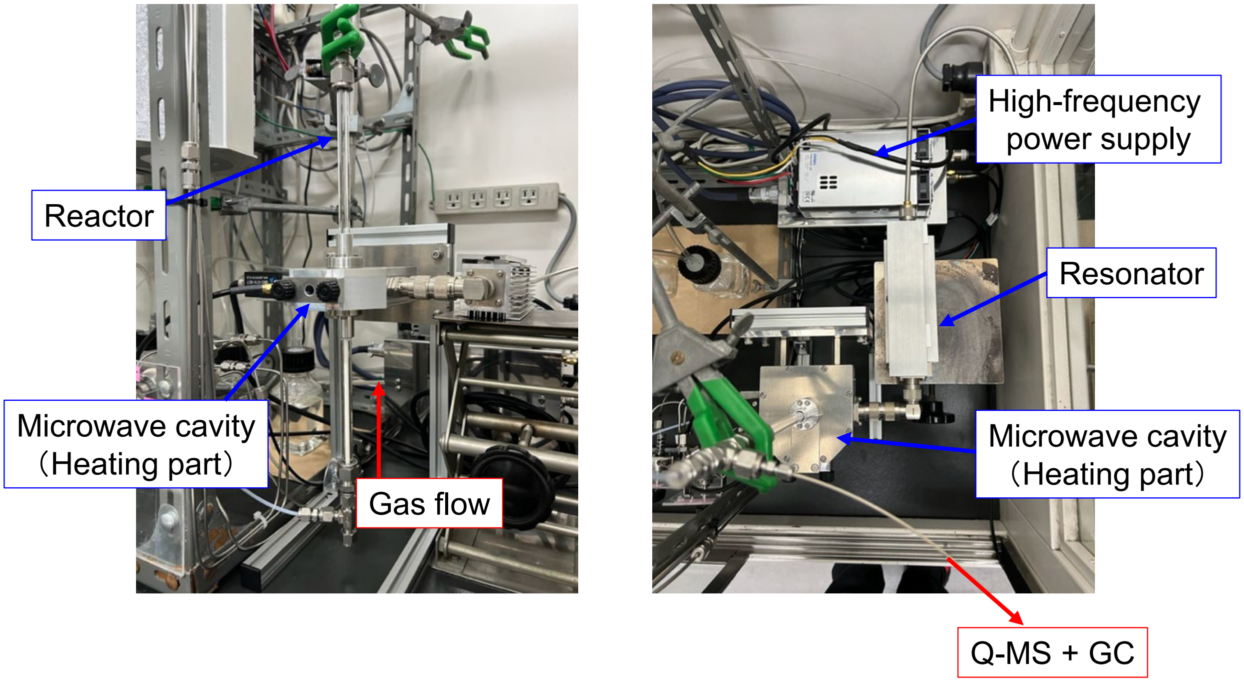

Figure S2. Photographs of the non-adiabatic reactor used for the triggering tests with microwave irradiation, related to STAR Methods.

(Left panel) Side view. (Right panel) Top view. The reactor was equipped with a microwave cavity, infrared thermometer, quadrupole mass spectrometer (Q-MS), and gas chromatograph (GC).

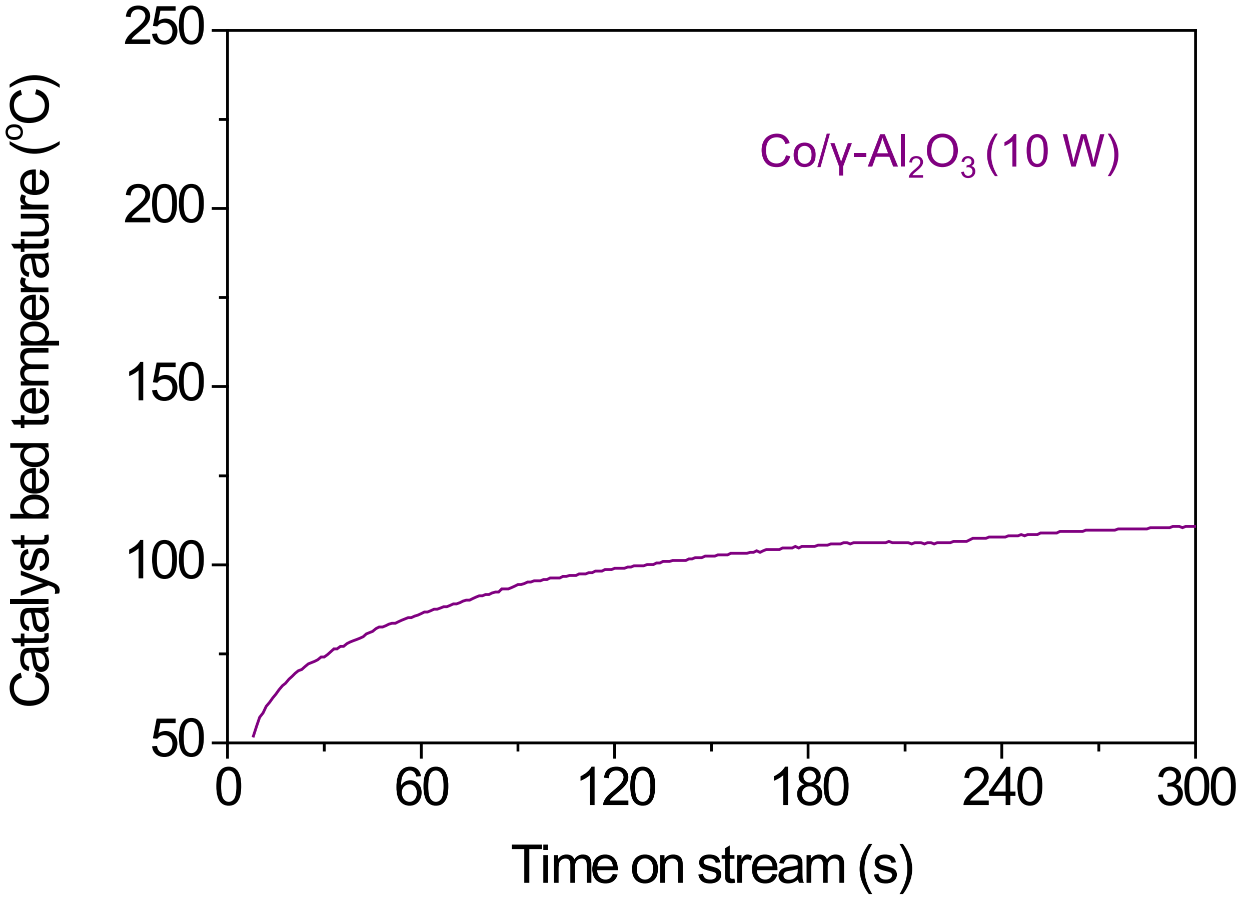

Figure S3. Time course of the catalyst bed temperature during the triggering test using 20 wt% Co/γ-Al_2O_3 and microwave irradiation at 10 W, related to Figure 2.

Oxidative decomposition of ammonia was not triggered within 300 s.

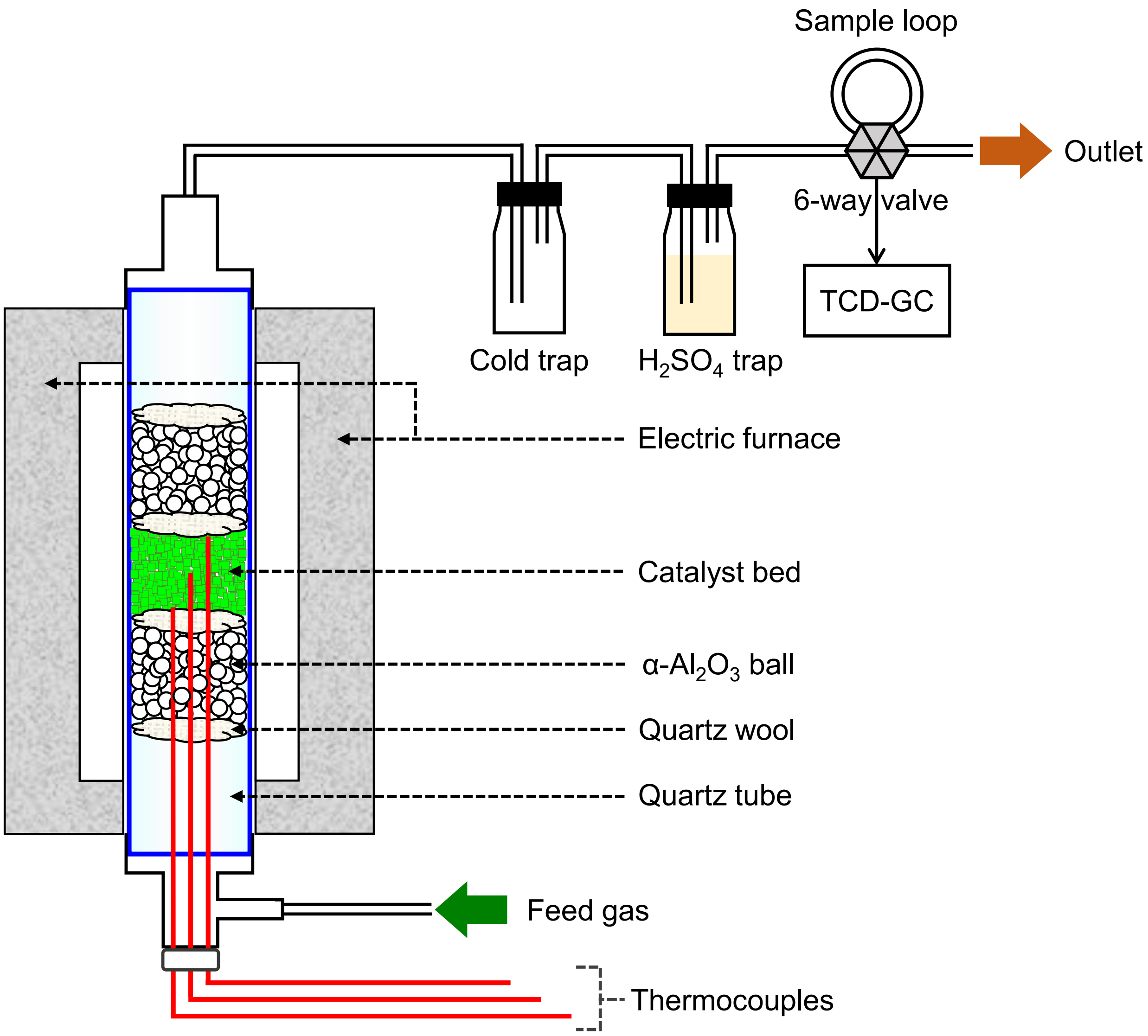

Figure S4. Schematic diagram of the quasi-adiabatic reactor used for the triggering tests using external heating by an electric furnace, related to Table 1.

The reactor was equipped with an electric furnace and gas chromatograph (GC) with a thermal conductivity detector (TCD). Three thermocouples were inserted at different positions in the catalyst bed.

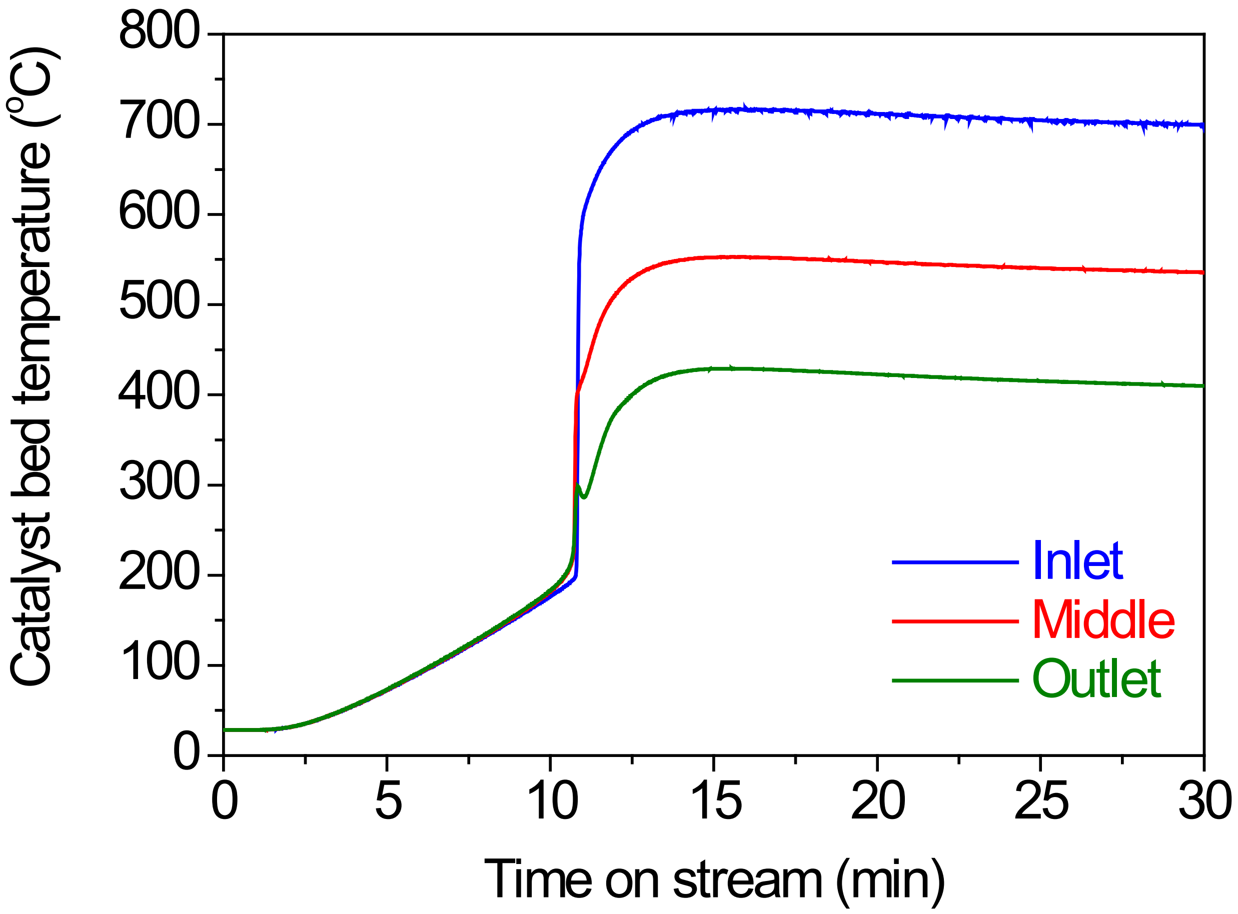

Figure S5. Time course of temperatures at three different positions in the catalyst bed during the triggering test using 20 wt% Co/$Ce_{0.5}Zr_{0.5}O_2$ and external heating by an electric furnace, related to Table 1.

The temperature was measured by thermocouples inserted at different positions in the catalyst bed. The heating was stopped at 11 min when the reaction was triggered.

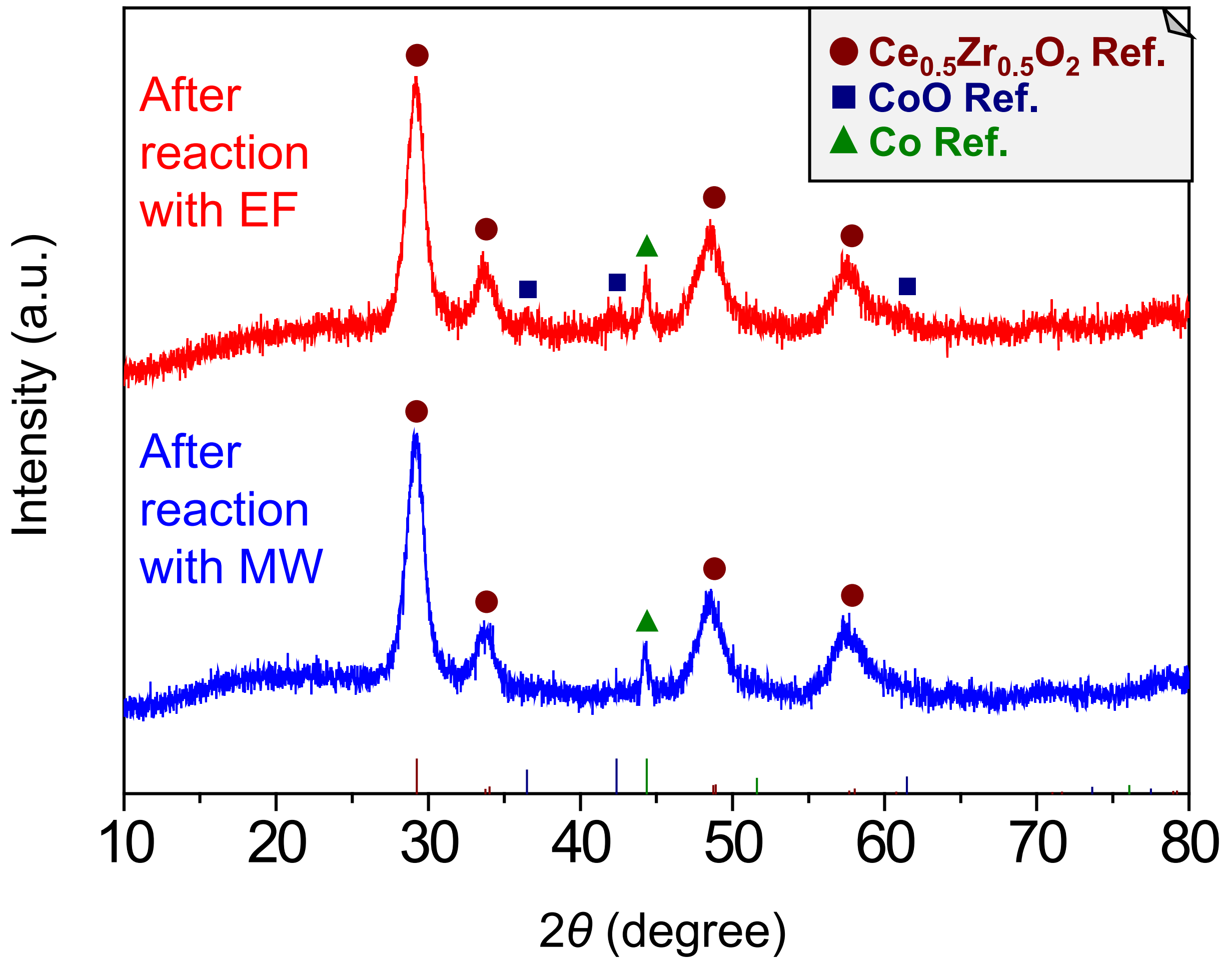

Figure S6. X-ray diffraction patterns of 20 wt% Co/$Ce_{0.5}Zr_{0.5}O_2$ catalyst recorded after oxidative decomposition of ammonia was triggered by heating by different method, related to Table 1.

EF: an electric furnace heating, MW: microwave irradiation. The reactions were halted by replacing the feed gas with helium.

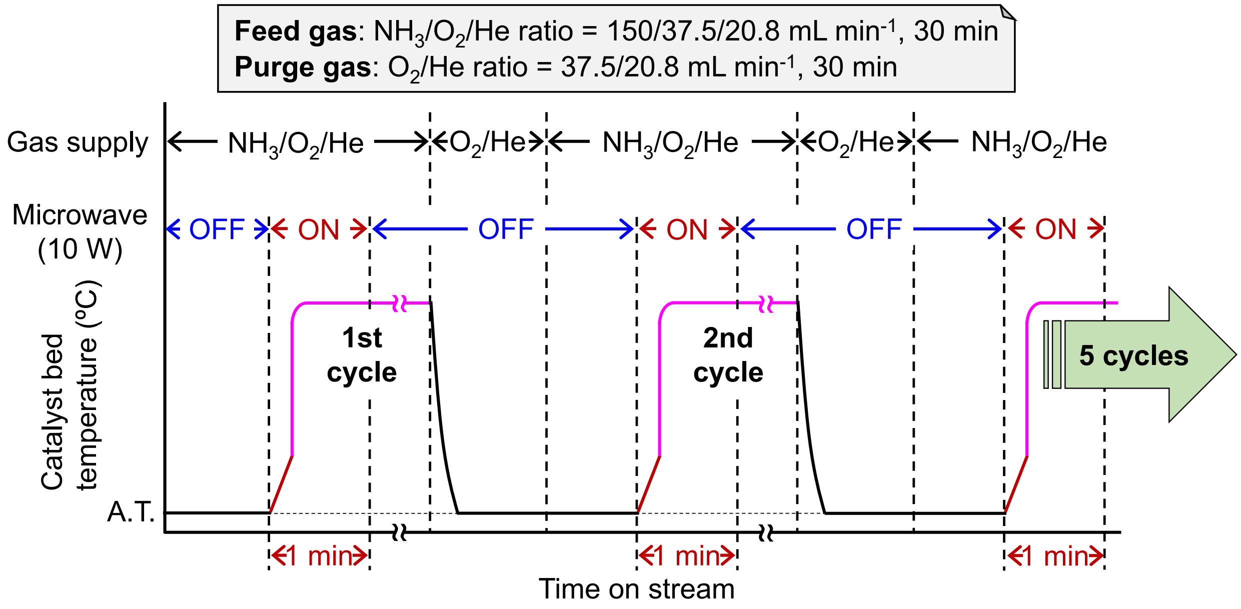

Figure S7. Experimental procedure used for the recycling test with microwave irradiation, related to Figure 7. After each cycle, the reactor was quenched to ambient temperature**.**

ON indicates microwave irradiation, OFF indicates no irradiation.

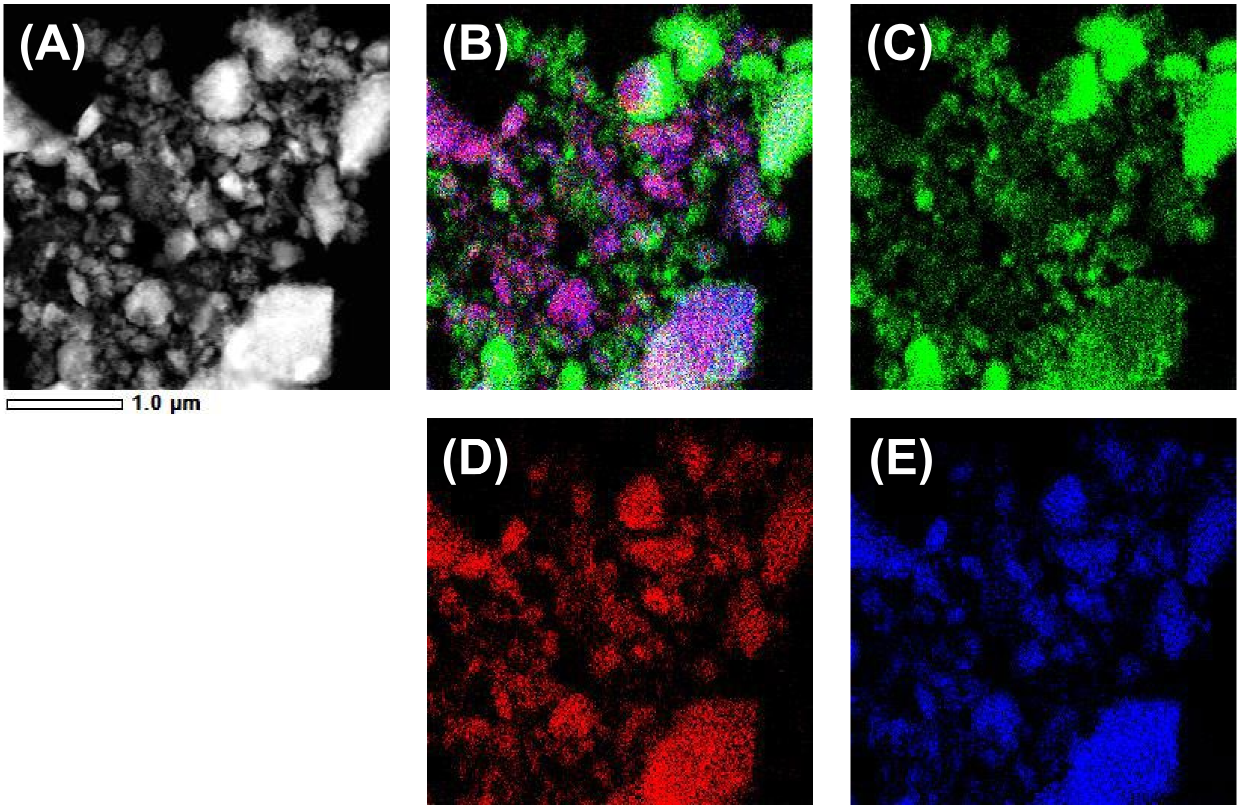

Figure S8. Electronic microscopic characterization of fresh 20 wt% Co/$Ce_{0.5}Zr_{0.5}O_2$, related to STAR Methods.

(A) High-angle annular dark-field scanning transmission electron microscopy image. (B–E) Energy dispersive X-ray elemental maps. (B) Overlay, (C) Co K-line, (D) Zr L-line, and (E) Ce L-line.

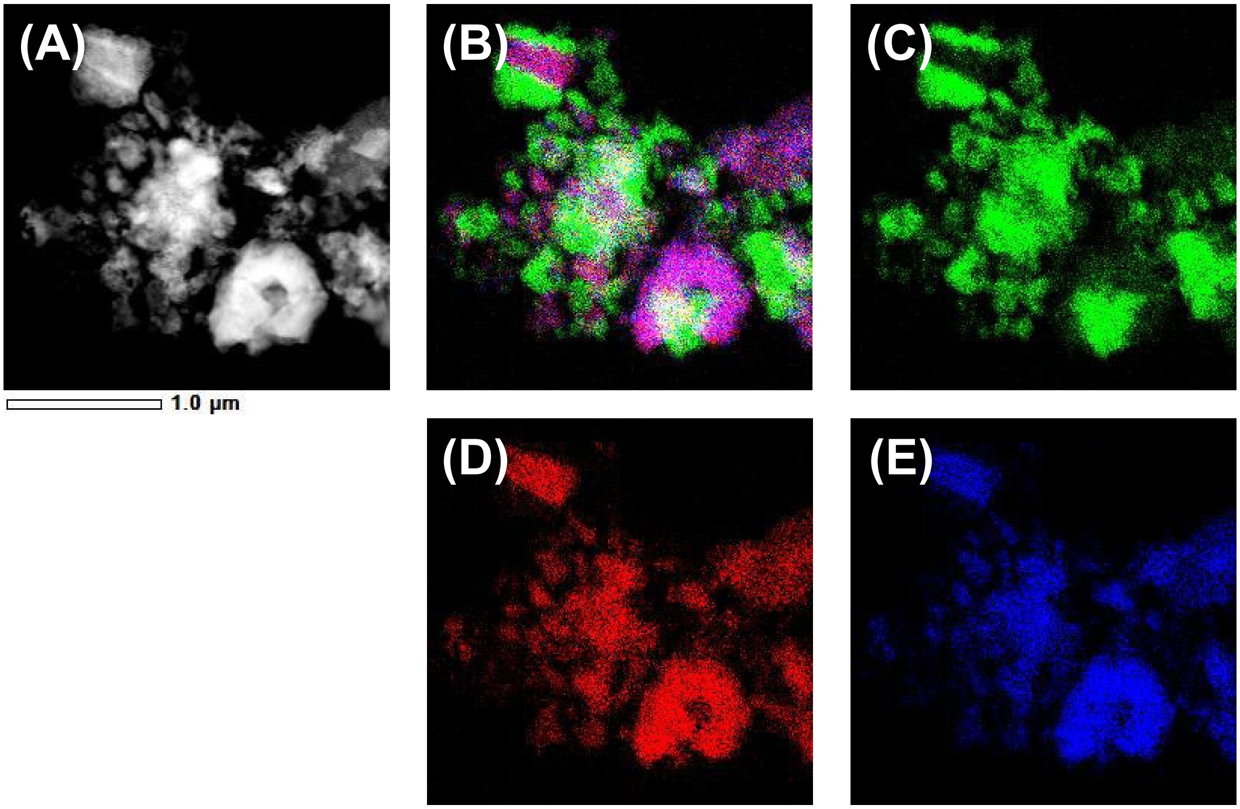

Figure S9. Electronic microscopic characterization of 20 wt% Co/$Ce_{0.5}Zr_{0.5}O_2$ after the fifth cycle of the recycling test, related to Figure 7.

(A) High-angle annular dark-field scanning transmission electron microscopy image. (B–E) Energy dispersive X-ray elemental maps. (B) Overlay, (C) Co K-line, (D) Zr L-line, and (E) Ce L-line.

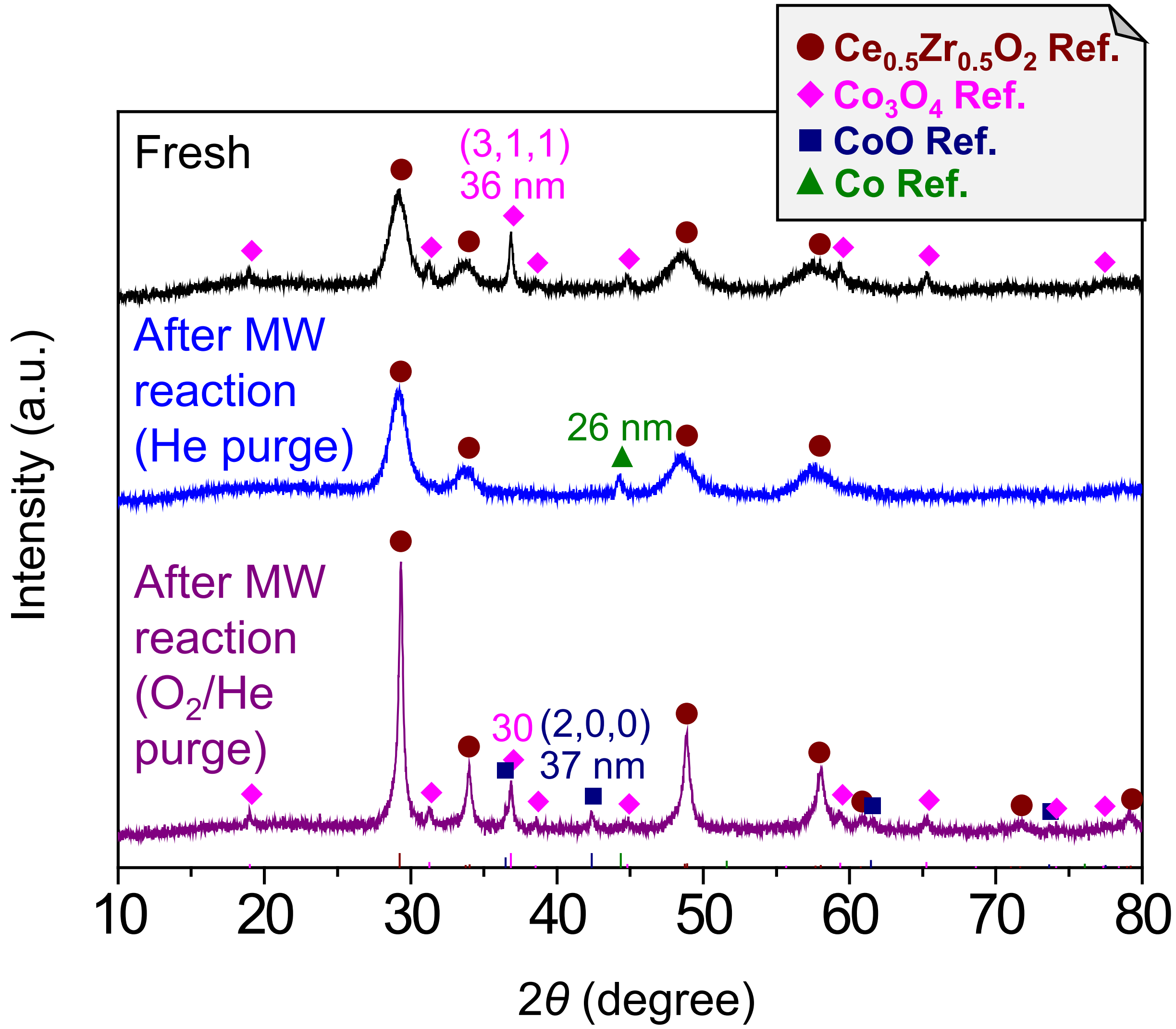

Figure S10. X-ray diffraction patterns of 20 wt% Co/$Ce_{0.5}Zr_{0.5}O_2$, related to Figure 7.

Patters were measured after cooling down to ambient temperature in He or an O_2/He mixture before the second cycle of the recycling test.

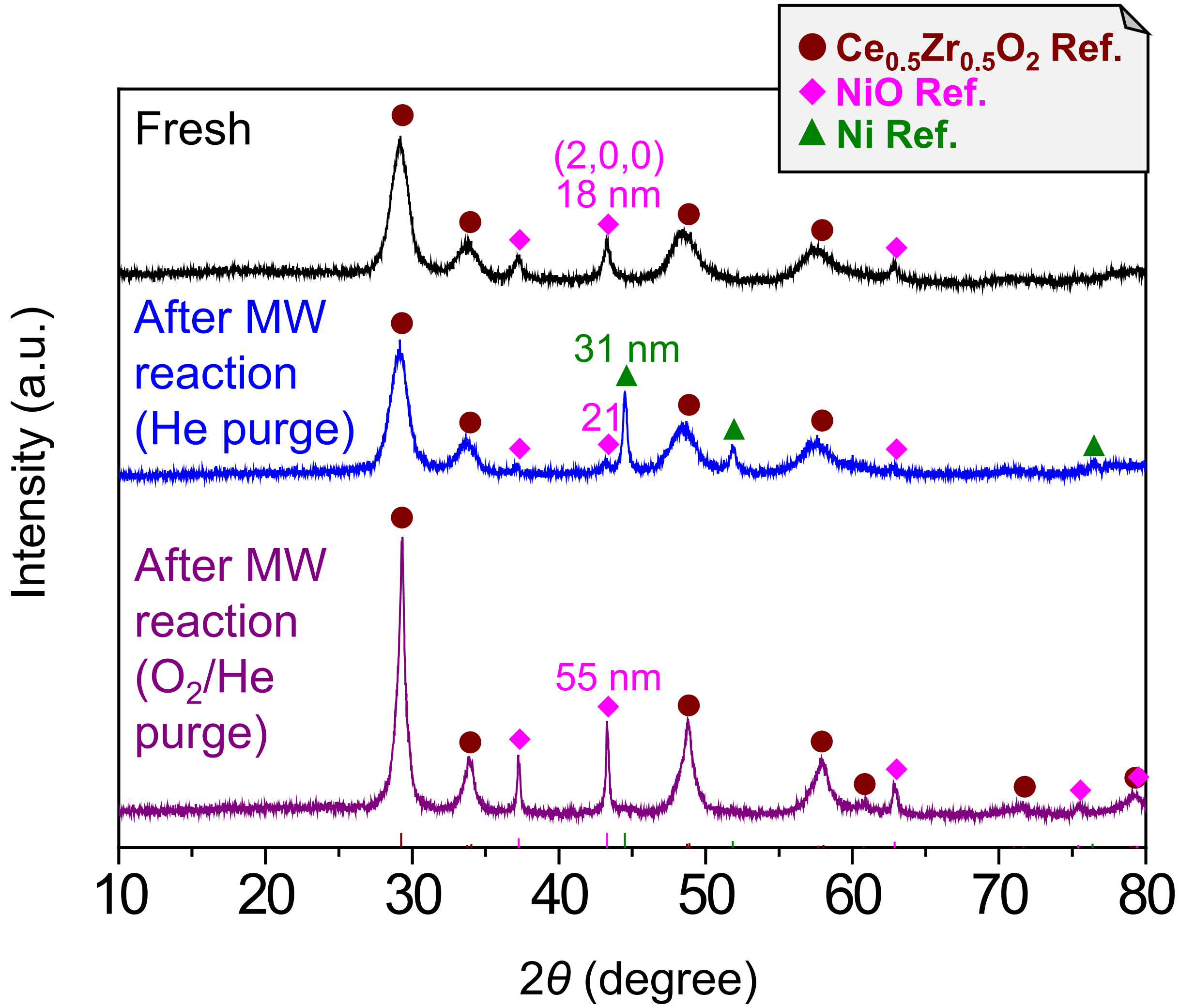

Figure S11. X-ray diffraction patterns of 20 wt% Ni/$Ce_{0.5}Zr_{0.5}O_2$, related to Figure 7.

Patters were measured after cooling down to ambient temperature in He or an O_2/He mixture before the second cycle of the recycling test.

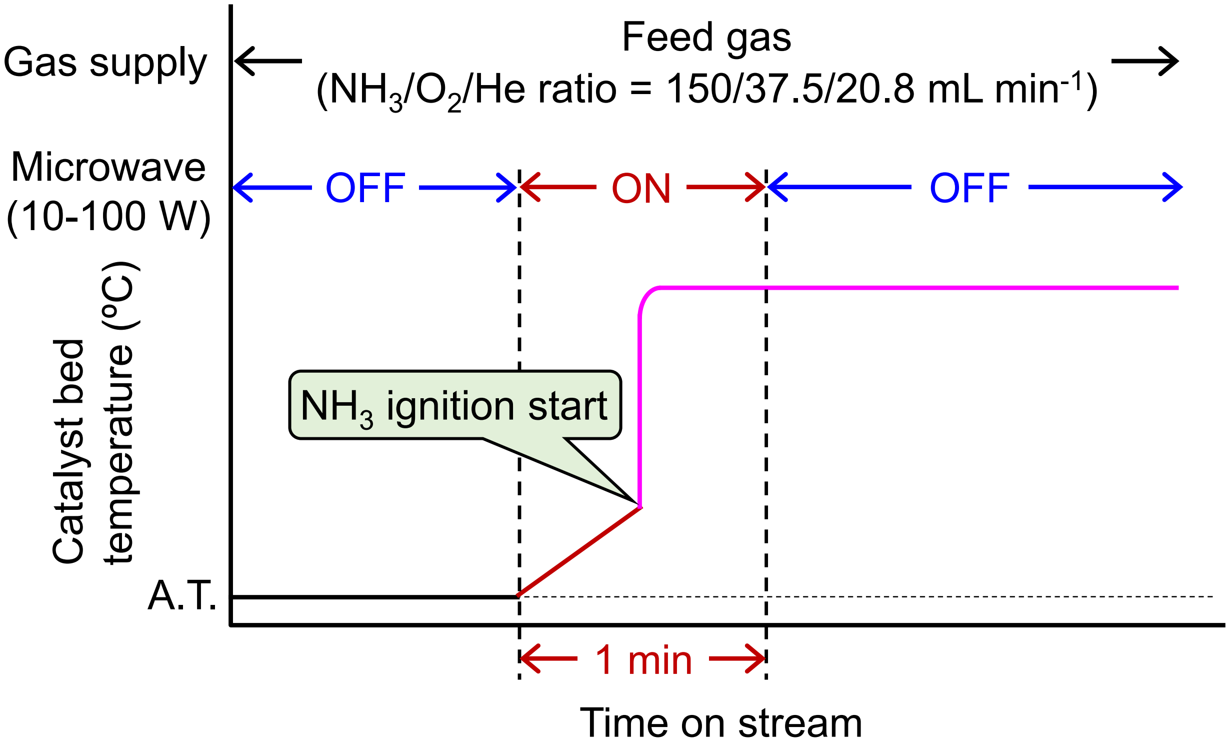

Figure S12. Standard experimental protocol for the triggering test with microwave irradiation, related to STAR Methods.

ON indicates microwave irradiation, OFF indicates no irradiation.

Calculation of NH_3 and O_2 conversions and H_2 yield

Due to the limitation of analysis by GC equipment, we could not directly estimate the composition of NH_3 in the effluent gas. Therefore, we calculated the NH_3 conversion based on the following method. The NH_3 conversion is described based on Equation S1.

$$NH_3 conversion\ (\%) = \frac{F_{NH_3-in} - F_{NH_3-out}}{F_{NH_3-in}} \times 100 \qquad \text{Equation S1}$$

where F_{in} and F_{out} are the molar rate of the gas species in inlet gas and effluent gas, respectively. In both cases of decomposition and combustion, 1 molecule of NH_3 is converted into 1/2 molecule of N_2. Thus, Equation S1 becomes Equation S2 as follows.

$$NH_3 conversion\ (\%) = \frac{F_{N_2-out} \times 2}{F_{NH_3-in}} \times 100 \qquad \text{Equation S2}$$

Here, the relationship between the flow rate and the concentration in the outlet gas for analysis by the GC is as follows.

$$F_{N_2-out} = \frac{C_{N_2-out-dry}}{C_{He-out-dry}} \times F_{He-out} \qquad \text{Equation S3}$$

$$\because F_{He-out} : F_{N_2-out} = C_{He-out-dry} : C_{N_2-out-dry}$$

where $C_{out\text{-}dry}$ are composition of effluent gas after passing through a cold trap to remove water and an H_2SO_4 trap. Since He is used as the internal standard, the flow rate of He in the inlet and effluent gas mixture is the same ($F_{He-out} = F_{He-in}$). Therefore, we can obtain NH_3 conversion based on the Equation S4 as follows.

$$NH_3 conversion\ (\%) = \frac{\left(\frac{C_{N_2-out-dry}}{C_{He-out-dry}} \times F_{He-in}\right) \times 2}{F_{NH_3-in}} \times 100 \qquad \text{Equation S4}$$

(eq. 4 in main manuscript)

In addition, with using similar way, we can calculate O_2 conversion and H_2 yield as follows.

$$O_2\ conversion\ (\%) = \frac{F_{O_2-in} - F_{O_2-out}}{F_{O_2-in}} \times 100 \qquad \text{Equation S5}$$

$$F_{O_2-out} = \frac{C_{O_2-out-dry}}{C_{He-out-dry}} \times F_{He-out} = \frac{C_{O_2-out-dry}}{C_{He-out-dry}} \times F_{He-in} \qquad \text{Equation S6}$$

$$\because F_{He-out} : F_{O_2-out} = C_{He-out-dry} : C_{O_2-out-dry}$$

$$\because F_{He-out} = F_{He-in}$$

$$O_2\ conversion\ (\%) = \frac{F_{O_2-in} - \left(\frac{C_{O_2-out-dry}}{C_{He-out-dry}} \times F_{He-in}\right)}{F_{O_2-in}} \times 100 \qquad \text{Equation S7}$$

(eq. 5 in main manuscript)

$$H_2\ yield\ (\%) = \frac{F_{H_2-out} \times 2}{F_{NH_3-in} \times 3} \times 100$$ Equation S8

$$F_{H_2-out} = \frac{C_{H_2-out-dry}}{C_{He-out-dry}} \times F_{He-out} = \frac{C_{H_2-out-dry}}{C_{He-out-dry}} \times F_{He-in}$$ Equation S9

$$\because F_{He-out} : F_{H_2-out} = C_{He-out-dry} : C_{H_2-out-dry}$$

$$\because F_{He-out} = F_{He-in}$$

$$H_2\ yield\ (\%) = \frac{\left(\frac{C_{H_2-out-dry}}{C_{He-out-dry}} \times F_{He-in}\right) \times 2}{F_{NH_3-in} \times 3} \times 100$$ Equation S10

(eq. 6 in main manuscript)
